# Diversity analysis of thermophilic hydrogenogenic carboxydotrophs by carbon monoxide dehydrogenase amplicon sequencing using new primers

**DOI:** 10.1007/s00792-020-01211-y

**Published:** 2021-01-07

**Authors:** Kimiho Omae, Tatsuki Oguro, Masao Inoue, Yuto Fukuyama, Takashi Yoshida, Yoshihiko Sako

**Affiliations:** 1 Laboratory of Marine Microbiology, Graduate School of Agriculture, Kyoto University, Kitashirakawa Oiwake-cho, Sakyo-ku, Kyoto, 606-8502 Japan; 2 Department of Biological Sciences, Graduate School of Science, The University of Tokyo, 2-11-16 Yayoi, Bunkyo-ku, Tokyo, 113-0032 Japan; 3 Research Center for Bioscience and Nanoscience, Japan Agency for Marine-Earth Science and Technology, 2-15 Natsushima-cho, Yokosuka, Kanagawa 237-0061 Japan

**Keywords:** Carbon monoxide dehydrogenase, Carboxydotroph, Hydrogen production, Group 4 [NiFe] hydrogenase, Thermophile, Amplicon sequencing, Rare biosphere

## Abstract

The microbial H_2_-producing (hydrogenogenic) carbon monoxide (CO)-oxidizing activity by the membrane-associated CO dehydrogenase (CODH)/energy-converting hydrogenase (ECH) complex is an important metabolic process in the microbial community. However, the studies on hydrogenogenic carboxydotrophs had to rely on inherently cultivation and isolation methods due to their rare abundance, which was a bottleneck in ecological study. Here, we provided gene-targeted sequencing method for the diversity estimation of thermophilic hydrogenogenic carboxydotrophs. We designed six new degenerate primer pairs which effectively amplified the coding regions of CODH genes forming gene clusters with ECH genes (CODHech genes) in Firmicutes which includes major thermophilic hydrogenogenic carboxydotrophs in terrestrial thermal habitats. Amplicon sequencing by these primers using DNAs from terrestrial hydrothermal sediments and CO-gas-incubated samples specifically detected multiple CODH genes which were identical or phylogenetically related to the CODHech genes in Firmictes. Furthermore, we found that phylogenetically distinct CODHech genes were enriched in CO-gas-incubated samples, suggesting that our primers detected uncultured hydrogenogenic carboxydotrophs as well. The new CODH-targeted primers provided us with a fine-grained (~ 97.9% in nucleotide sequence identity) diversity analysis of thermophilic hydrogenogenic carboxydotrophs by amplicon sequencing and will bolster the ecological study of these microorganisms.

**Supplementary Information:**

The online version contains supplementary material available at 10.1007/s00792-020-01211-y.

## Introduction

Hydrogenogenic carboxydotrophs, a type of anaerobic microorganism, grow by coupling the oxidation of carbon monoxide (CO) with the production of H_2_ (Robb and Techtmann 2018). While CO is present in a wide variety of environments due to natural chemical and biological processes (Conrad 1996; Conte et al. 2019; Khalil and Rasmussen 1990; King and Weber 2007; Mörsdorf et al. 1992), it is toxic to many microorganisms and inhibits growth (Carvalho et al. 2019; Davidge et al. 2009; Davidova et al. 1994; Nobre et al. 2007; Parshina et al. 2005a; Tavares et al. 2011). Therefore, it is predicted that the CO-dependent H_2_ production of hydrogenogenic carboxydotrophs is an important metabolic mechanism by which toxic CO is reduced and H_2_, an energy source for H_2_-utilizing microbial communities, is generated (Techtmann et al. 2009). The CO-oxidizing and H_2_-producing system involve the coupling of CO dehydrogenases (CODHs) and membrane-bound H_2_-evolving Group 4 [NiFe] hydrogenases which are called energy-converting hydrogenases (ECHs) (Søndergaard et al. 2016; Techtmann et al. 2012). CODH and ECH genes are often found in a single gene cluster (CODH–ECH gene cluster) in the hydrogenogenic carboxydotroph (Inoue et al. 2019; Sokolova and Lebedinsky 2013; Techtmann et al. 2012), and are predicted to encode a CODH/ECH complex, which, along with an ATP synthase, might comprise the machinery for simple respiration (Schoelmerich and Müller 2019).

Today, 37 hydrogenogenic CO-oxidizing isolates have been reported in five phyla, 20 genera, and 32 species from various settings, including marine environments (deep-sea hydrothermal vent and marine sediment), terrestrial aquatic environments (hot spring and fresh water), soils, and bioreactors (Fukuyama et al. 2020). Except for seven mesophilic bacteria of the phylum Proteobacteria, all hydrogenogenic CO-oxidizing isolates of the other four phyla (Firmicutes, Euryarchaeota, Crenarchaeota and Dictyoglomi) are (hyper-)thermophilic. In addition to their isolation and identification, several ecological studies on thermophilic hydrogenogenic carboxydotrophs have been conducted using radioisotopes (Kochetkova et al. 2011), quantitative polymerase chain reaction (qPCR) analysis targeting the CODH gene (Yoneda et al. 2015), and stable isotope probing (SIP) method by ^13^CO DNA (Brady et al. 2015) in terrestrial hydrothermal environments. These studies have suggested that diverse thermophilic hydrogenogenic carboxydotrophs occur widely in those environments. However, these methods had a few limitations to reveal the ecology of these microorganisms. For example, the previous qPCR method can amplify only the specific CODH genes (*cooS-II*) of *Carboxydothermus* species (Yoneda et al. 2015). The CO-SIP method detects CO-utilizing microorganisms which incorporates ^13^CO-carbon into their DNA (Brady et al. 2015), but it cannot evaluate heterotrophic hydrogenogenic CO-oxidizers like *Parageobacillus thermoglucocidasius* which seems to utilize CO as only an energy source (Mohr et al. 2018). To avoid taxonomic and cultivation bias in the exploration of thermophilic hydrogenogenic carboxydotrophs, culture-independent techniques like metagenomics are desired. But it seems that the rare relative abundance of these microorganisms (Brady et al. 2015; Yoneda et al. 2015) makes it difficult to recover CODH–ECH gene clusters containing metagenome-assembled genomes from environments.

Amplicon sequencing which reveals the community structure in high-resolution with deeply sequenced target genes is another option for diversity estimation of thermophilic hydrogenogenic carboxydotrophs. A recent bioinformatics-based analysis identified 71 genomes harboring the CODH–ECH gene clusters from ~ 140,000 prokaryotic genomes analyzed, which expanded the diversity estimation of potential hydrogenogenic carboxydotrophs to four phyla, 26 genera, and 43 species and provided the reference for amplicon sequencing (Omae et al. 2019). By using the 16S rRNA gene sequences retrieved from these 71 genomes as a reference in microbial community analysis, we revealed that multiple species of potential thermophilic hydrogenogenic carboxydotrophs in the phylum Firmicutes were distributed in a wide variety of terrestrial hydrothermal environments in culture-independent way (Omae et al. 2019). However, we were unable to define hydrogenogenic carboxydotrophs by the 16S rRNA gene sequence alone, because strains with and without hydrogenogenic carboxydotrophy exist within a single species (Omae et al. 2019). Additionally, phylogenetically novel hydrogenogenic carboxydotrophs may remain unidentified by the 16S-targeted amplicon sequencing based on the 71 reference genomes. As such, amplicon sequencing by primers that target the CODH gene of the CODH–ECH gene cluster (hereafter called “CODHech gene”) may be effective for identification of novel hydrogenogenic carboxydotrophs.

However, a high diversity of CODHs prevents the design of a universal primer for PCR amplification. Phylogenetically, the ~ 2000 CODH genes currently described in genomic sequence databases fall into seven clades (Clades A–G) (Inoue et al. 2019). However, as is the case in CODHech genes, the function of a given CODH is often predicted based on other genes in close proximity (i.e. genomic context) (Inoue et al. 2019; Techtmann et al. 2012). For example, a CODH within an acetyl coenzyme A (acetyl-CoA) synthase (ACS) gene cluster is predicted to fix CO_2_ via the Wood-Ljungdahl pathway (Ragsdale 2004), while a CODH adjacent to a CooF gene, which encodes a ferredoxin-like electron carrier, is predicted to oxidize CO and transfer electrons to the CooF, thereby producing reducing power (Fox et al. 1996). Functionally similar CODHs are often found within phylogenetically different clades. For example, CODHs in CODH–ACS, CODH–CooF, and CODH–ECH gene clusters are found in Clades A/E/F, Clades C/E/F and Clades E/F, respectively (Inoue et al. 2019; Techtmann et al. 2012). Meanwhile, CODH genes within a single clade that share functionality often form monophyletic subclades. It is suggested that both horizontal gene transfer and vertical transmission have driven the remarkable divergence of CODHs (Techtmann et al. 2012).

In the present study, to address the diversity of hydrogenogenic carboxydotrophs, we designed multiple degenerate primers which effectively amplified CODHech genes, enabling amplicon sequencing that target those species. To cover the major taxa of thermophilic hydrogenogenic carboxydotrophs which are predicted to be widely distributed across environments (Fukuyama et al. 2020; Omae et al. 2019), the new primers were designed to amplify the CODHech genes identified in Firmicutes (hereafter called “FirmiCODHech genes”). The FirmiCODHech genes were derived from 20 species within 12 genera, including one uncultivated strain, and formed three subclades within Clades E and F. Amplicon sequencing by these primers in hot spring sediment samples with or without incubation under CO gas successfully identified phylogenetically novel CODHech genes which may be derived from uncultured hydrogenogenic carboxydotrophs, as well as those of known species.

## Materials and methods

### Identification and classification of CODHech genes

The 71 genomes harboring the CODH–ECH gene clusters were identified from ~ 140,000 prokaryotic genomes in the Reference Sequence (RefSeq) Database in the National Center for Biotechnology Information (NCBI) (December 2018) (NCBI Resource Coordinators 2018) as previously described (Omae et al. 2019). In addition, we performed phylogenetic analyses on CODH and ECH catalytic subunit genes for classification of these CODH–ECH gene clusters and primer design. The 1558 CODH proteins including 47 encoded in the 71 CODHech genes were obtained as previously described (Omae et al. 2019). We curated the CODH-encoding genomes from the NCBI assembly database (December 2018) by searching ‘feature_table’ for CODH protein accessions (NCBI Resource Coordinators 2018), which identified 5311 CODH genes in 3050 prokaryotic genomes. The CODH proteins were aligned with the MAFFT version 7.402 using the E-INS-I method (Katoh and Standley 2013). The multiple sequence alignment (MSA) was subsequently trimmed using the trimAl version 1.4.1 program with a gap-threshold value of 0.9 (Capella-Gutiérrez et al. 2009). A phylogenetic tree was then constructed using FastTree version 2.1.11 (Price et al. 2010) with an approximate-maximum-likelihood method using the WAG model. Robustness of the topology of the phylogenetic trees was evaluated by local bootstrap values based on 1000 re-samples. Phylogenetic classification of CODHs was performed as described in previous studies (Inoue et al. 2019). The tree was visualized using iTOL version 5.2 (Letunic and Bork 2016).

For retrieval of ECH genes, the amino acid sequences of Group 4 [NiFe]-hydrogenase catalytic subunit homologs were obtained from the RefSeq Database in NCBI (December 2018) through a BLASTp search (*E* value ≤ 0.001) in the BLAST + using the following representative proteins in HydDB (Søndergaard et al. 2016) as queries: *Escherichia coli* HycE (WP_014639275.1, Group 4a), *E. coli* HyfG (WP_014641051.1, Group 4a), *Pyrococcus abyss* MchD (WP_010868591.1, Group 4b), *Carboxydothermus hydrogenoformans* CooH (WP_011344721.1, Group 4c), *Pyrococcus furiosus* MbhL (WP_011012581.1, Group 4d), *Methanosarcina barkeri* EchA (WP_011305188.1, Group 4e), *Desulfosporosinus orientis* EhfE (WP_014183752.1, Group 4f), *Thermosphaera aggregans* MahB (WP_013129492.1, Group 4 g), *Methanothermobacter marburgensis* EhaO (WP_013295617.1, Group 4 h) and *Methanothermobacter marburgensis* EhbN (WP_013296415.1, Group 4i). The obtained protein sequences were classified using a HydDB classifier to select Group 4 [NiFe]-hydrogenase (Søndergaard et al. 2016). Further quality control was performed by MSA using the MAFFT with the FFT-NS-2 method (Katoh and Standley 2013), discarding sequences which lacked conserved cysteine residues required to ligate H_2_-binding metal centers (L1 and L2 motifs) (Vignais and Billoud 2007). We curated the Group 4 [NiFe]-hydrogenase catalytic subunit-encoding genomes as described above, resulting in 3464 Group 4 [NiFe]-hydrogenase catalytic subunit proteins encoded in 50,441 genes of 38,046 prokaryotic genomes. A phylogenetic tree was constructed as described above with modification, where MSA was performed by MAFFT with the FFT-NS-2 method and trimming and tree-construction were performed with the default settings of trimAl and FastTree, respectively.

### Design of new CODH-targeted primers

Of the 71 CODHech genes identified, 34 were derived from Firmicutes members. These 34 FirmiCODHech nucleotide sequences were aligned with MAFFT and the conserved genetic regions were visualized by calculating the average ratio of the dominant base to all bases at each position in a 20-base sliding window. We designed six primer sets for each target group, whose specificity was checked by in silico PCR using the ‘primersearch’ program in the EMBOSS version 6.6.0 package allowing 10% mismatch (Rice et al. 2000) (Table 1).

**Table 1:** List of the primer sets designed in this study to amplify Firmicutes CODHech genes

Primer set name	Target clade	Fw primer sequence (5′—> 3′)	Rv primer sequence (5′—> 3′)	Degeneracy	Expected amplicon size (bp)
E4a_p1	E4a	CCCAGAGCTTGAAGCTTTAGCC	CTACTAGCGCCGCTATACCAC	1	500
F4a_p1	F4a	GTGGTRGGCATCTGCTGYAC	GCGKRAYCTTGACGTTRTTGCA	64	490
F4a_p2	F4a	TGGATTACCAGTGCATCATGCCC	RAACCCGTGGCGCATGAGC	2	473
F4c1_p1	F4c1	GTCGTATYGATCCWTTTGGCAATGG	KTATAATCRGCMAGTGCTCCCTTTA	32	502
F4c1_p2	F4c1	GGSGTGCTGAAGGAAGATGC	RATTGCCTCRGCACTGAAMC	16	501
F4c2_p1	F4c2	GATGCWCAYACCATTGTGGCG	ATAATTCGGTWGACAAATACATTCCGGT	8	478

### Determination of an optimal CODHech gene identity threshold for species classification

To determine the optimal threshold in CODHech gene sequence identity for species classification, we calculated precision-recall and F-measure (Kim et al. 2014) using taxonomic information and pairwise sequence identities of the 71 full-length CODHech gene sequences, and selected the identity threshold with the highest F-measure as the optimal species cut-off. First, we assigned the taxonomic information of the Genome Taxonomy Database (GTDB) release 89 (Parks et al. 2018), a standardized microbial taxonomy based on genome phylogeny, to the 71 genomes harboring CODH–ECH gene clusters. When no GTDB entry was found, genomes were assigned to the GTDB taxonomy by using GTDB-Tk version 2.2 (Chaumeil et al. 2020) . For the four genomes of Rhizobiales bacteria without species-level taxonomic information, the average nucleotide identity (ANI) between the genomes was calculated by using FastANI version 1.1 (Jain et al. 2018), and the genomes with < 95% ANI, which is a typical species ANI circumscription (Chaumeil et al. 2020), were assigned as the same species. Full-length pairwise sequence identity was computed for each pair of the 71 CODHech gene sequences using needleall in EMBOSS (-gapopen 10.0 -gapextend 0.5) (Rice et al. 2000), and the resulting value was noted along with the taxonomic relationship between the sequences, i.e., intra- or interspecies. A grid search approach was implemented to test all possible cutoff values between 80 and 100% nucleotide sequence identity with a step-size of 0.1%. For each possible cutoff value, the number of sequences that were correctly [true positives (TP) and true negatives (TN)] and incorrectly [false positives (FP) and false negatives (FN)] placed were computed in species-level comparisons. Precision and recall values were calculated as follows: precision = TP/(TP + FP) and recall = TP/(TP + FN). These values were then used to calculate the F-measure, which is a harmonic mean of precision and recall and represents the accuracy of the test. We performed the same analysis with the data set which were trimmed into the amplification regions of each primer set.

### Collection of environmental samples

Two sediment samples of thermophilic environments, UN and JI, were collected from the terrestrial hot springs of Unagi-onsen (temperature, 46.4 °C; pH 2.9; ORP, 487 mV) located in Kagoshima Prefecture (31°13′41″N., 130°36′47″E), Japan, and Jiunji-onsen (temperature, 60.1 °C; pH 7.7; ORP, 259 mV) in Shizuoka Prefecture (34°38′54″N., 138°52′00″E), Japan, in December 2012 and January 2015, respectively. One sediment sample from a mesophilic environment, which was used to prepare a CODHech-mock community sample, was collected from Unagi-ike lake (temperature, 22.0 °C; pH 7.9; ORP, 446 mV) located in Kagoshima Prefecture (31°13′39″N., 130°36′35″E), Japan, in May 2018. The temperatures, pH, and ORP of the sediment pore water were measured as previously described (Omae et al. 2019). Samples were then packed in a cooler with ice, transported to the laboratory, and stored at − 80 °C until use.

### Enrichment of thermophilic hydrogenogenic carboxydotrophs

To enrich and analyze rare thermophilic hydrogenogenic carboxydotrophs, the sediment of JI was subjected to incubation under 10% CO at 65 °C. Approximately 5 ml of JI sediment and pore water in total was placed in two glass vials (64 ml), which were then sealed with butyl rubber stoppers. The gas phase of each vial was replaced by CO and N_2_ at mixing rations of 10% v/v CO, and the vials were vigorously vortexed. After five days’ incubation at 65 °C, each enriched sample was collected and stored at –80 °C until DNA extraction. The H_2_ production in each vial after five days’ incubation was checked by using a GC-2014 gas chromatograph (Shimadzu, Kyoto, Japan) equipped with a thermal conductivity detector and a ShinCarbon ST packed column (Shinwa Chemical Industries, Kyoto, Japan). Argon was used as the carrier gas.

### Preparation of the CODHech-mock community sample

To evaluate the specificity and quantitativity of the new CODH-targeted primer sets, we prepared a mock community sample containing the cells of four species of thermophilic hydrogenogenic carboxydotrophs of the phylum Firmicutes, which harbor the F4c1 or F4a CODHech genes as follows: *C. hydrogenoformans* Z-2901, CHY_RS08505 (F4c1); *Carboxydocella* sp. ULO1, ULO1_RS08880 (F4c1); *Calderihabitans maritimus* KKC1, KKC1_RS06675 (F4c1); *P. thermoglucosidasius* DSM 2542, AOT13_RS13420 (F4a). The culture of *C. hydrogenoformans* Z-2901 (= DSM 6008^T^) was purchased from Deutsche Sammlung von Mikroorganismenund Zellkulturen (DSMZ). The culture of *P. thermoglucosidasius* DSM 2542 (= NBRC107763^T^) was purchased from the Biological Resource Center, National Institute of Technology and Evaluation (NBRC). Cells of *Carboxydocella* sp. ULO1 and *C. maritimus* KKC1 were isolated and maintained in our laboratory (Fukuyama et al. 2017; Yoneda et al. 2013). The cells of *C. hydrogenoformans* Z-2901 and *Carboxydocella* sp. ULO1 were grown in a modified DSM 507 medium (pH 7.0) (Fukuyama et al. 2018), and the cells of *C. maritimus* KKC1 were grown in a NBRC 1251 medium (pH 7.5). These were grown under 100% CO gas at 65 °C by using 100 ml butyl rubber-stoppered bottles containing 50 ml of medium. The cells of *P. thermoglucosidasius* DSM 2542 were grown in a NBRC 802 medium under the aerobic condition at 65 °C and 100 rotations per minute (rpm) using a shaking Erlenmeyer flask containing 100 ml of medium. Rinsed cells of the four species were resuspended in filter-sterilized water containing 8 g l^−1^ of NaCl, mixed and added to 2 g of the mesophilic sediment sample which was collected from Unagi-ike lake as mentioned above. The sample was stored at –80 °C until DNA extraction.

### DNA extraction

DNA was extracted from 0.5 g of the samples using an Extrap Soil DNA Kit Plus ver. 2 (Nippon Steel and SUMIKIN Eco-Tech, Tokyo, Japan) following the manufacturer’s instructions. During the homogenizing step, we used a bead beater-type homogenizer, Beads Crusher μT-12 (Taitec, Saitama, Japan), at a speed of 3200 rpm for 60 s. The extracted DNA was stored at − 30 °C until use.

### Quantification of CODHech genes

To reveal microbial composition, the CODHech gene sequences of *C. hydrogenoformans* Z-2901, *Carboxydocella* sp. ULO1, *C. maritimus* KKC1, and *P. thermoglucosidasius* DSM 2542 in the CODHech-mock community sample were quantified by qPCR (Online Resource Table S1). Specificity of each designed qPCR primer set was checked by Primer-BLAST (Ye et al. 2012). The reaction mixture contained 2 μL of the CODHech-mock community DNA template with 12.5 μL of TB Green *Premix Ex Taq* II (Tli RNaseH Plus) (TaKaRa Bio, Shiga, Japan), according to the manufacturer’s instructions. PCR amplification was performed using the Thermal Cycler Dice real-time system TP850 (TaKaRa Bio). The cycling programs were as follows: 1 min at 95 °C for initial denaturation; 38 cycles of 5 s at 95 °C; 10 s at 55 °C for CHY_RS08505 and ULO1_RS08880, 58 °C for KKC1_RS06675, or 60 °C for AOT13_RS13420; and 20 s at 72 °C. Disassociation curves were created by gradually increasing the temperature from 60 to 95 °C after PCR to verify amplification specificity. The qPCR standard curve for each targeted gene showed a log-linear relationship when a tenfold dilution series of PCR products (from 10^1^ to 10^7^ copies μl^−1^). All qPCR data represent the mean values of triplicate technical determinations.

### PCR amplification and sequencing

The primer sets F4a_p1, F4c1_p1, and F4c1_p2 were used for the CODHech-mock community DNA template, while all primer pairs were used for environmental and enrichment samples. Overhang adapters were appended at the 5′ end of each primer (forward overhang: 5′-TCGTCGGCAGCGTCAGATGTGTATAAGAGACAG, reverse overhang: 5′-GTCTCGTGGGCTCGGAGATGTGTATAAGAGACAG) according to the Illumina 16S Metagenomic Sequencing Library Preparation guide (https://support.illumina.com/content/dam/illumina-support/documents/documentation/chemistry_documentation/16s/16s-metagenomic-library-prep-guide-15044223-b.pdf). The PCR reaction mixture contained 12.5 μL 2 × KAPA HiFi HotStart ReadyMix (5 mM Mg^2+^) (KAPA BIOSYSTEMS, Wilmington, MA, USA), 2.5 μL DNA template, and 5 μL of each primer (10 μM) for final volumes of 25 μL. PCR was performed in “touch down” mode: initial denaturation at 95 °C for 3 min; 10 cycles of 30 s denaturation at 95 °C, 30 s annealing at 69–59 °C (temperature decreased by 1 °C per cycle during the first 10 cycles) and elongation for 30 s at 72 °C; 28 cycles of 30 s denaturation at 95 °C, 30 s annealing at 59 °C and elongation for 30 s at 72 °C; final elongation at 72 °C for 5 min. The resulting PCR products were examined in 1.5% (w/v) agarose electrophoresis in 1 × Tris–acetate EDTA buffer and stained with 3 × GelGreen Nucleic Acid Gel Stain (Biotium, Fremont, CA, USA). Bands of expected sizes were visualized on the Visi-Blue Transilluminator (UVP, Upland, CA, USA), excised and purified with the Wizard SV Gel and PCR CleanUp System (Promega, Madison, WI, USA). To distinguish reads from different PCR products, multiplex barcodes were attached to the amplicons according to Illumina’s 16S library preparation guide. DNA concentration of the library was determined by Qubit HS dsDNA Assay Kit (Thermo Fisher Scientific, Waltham, MA, USA). The molarity was calculated according to Illumina’s 16S library preparation guide. All amplicons were diluted to 1 nM and mixed. Further dilution yielded 12 pM final libraries. The sequencing was performed using the Illumina MiSeq platform with MiSeq V3 (2 × 300 bp) reagent kits (Illumina, San Diego, CA, USA) and with a spike-in of PhiX at 30% to serve as an internal control.

### Sequence data processing and analyses

Adapter and primer-binding regions were trimmed from the 5′ ends of the forward and reverse reads with the VSEARCH version 2.14.1 (Rognes et al. 2016). The reads were further processed by trimming low-quality regions from the sequences with the Trimmomatic version 0.36 program (SLIDINGWINDOW: 50:20) (Bolger et al. 2014). Using VSEARCH, the paired-end reads were joined, and a further round of quality control (QC) was conducted to remove sequences shorter than 200 nt as well as those containing ambiguous bases (N) or bases with a quality score below 20 (--fastq_mergepairs --fastq_minmergelen 200 --fastq_maxns 0 --fastq_qminout 20). The merged sequences were pooled at each primer set and dereplicated to unique sequences by VSEARCH (--derep_fulllength). We performed stringent denoising strategy by the UNOISE3 algorithm (Edgar 2016) implemented within VSEARCH to cluster the remaining sequences into operational taxonomic units (OTUs), where unique sequences with abundances of < 8 were discarded (--cluster_unoise --id 0.979 --minsize 8 --unoise_alpha 2.0). In OTU clustering, we applied 97.9% nucleotide identity threshold for species classification, which was determined as described in the previous section. Chimeric sequences were removed by the UNOISE3 algorithm implemented within VSEARCH (--uchime3_denovo –abskew 16). OTUs derived from the CODH genes were selected by searching the CODH protein dataset using DIAMOND version 0.9.22 (Buchfink et al. 2015), where OTUs with bit score < 80 were discarded. Furthermore, the OTUs which were aligned within each primer target region were selected as ‘CODH-OTUs’. For phylogenetic classification, the resulting CODH-OTU amino acid sequences were added to the existing multiple sequence alignment of the CODH proteins by MAFFT with the E-INS-i method. A phylogenetic tree was constructed and visualized as described above.

### Determination of the genomic context of the detected CODH-OTUs

While the amplification of the CODH-OTUs strongly suggests the presence of hydrogenogenic carboxydotrophs, we cannot determine that the amplified CODH genes truly form gene clusters with ECH genes. To verify that the CODH genes detected in this study formed gene clusters with ECH genes, we amplified and sequenced the genetic region between the CODH-OTUs and associated ECH genes from the CO-enriched samples by combining the new CODH-targeted primers and ECH-targeted primers. The ECH-targeted primers, ECH4a_Rv (5′-CCGTCGACAWAGAGYCGGAA) and ECH4c_Fw (5′-CCCTTYACYTAYTGYATGGC), were constructed at conserved sites of the ECH (Group 4 [NiFe]-hydrogenase) genes associated with FirmiCODHechs of subclades F4a and F4c, respectively (Online Resource Table S2). Tks Gflex DNA Polymerase (TaKaRa Bio) was used for PCR. The PCR mixtures (25 µl) contained 12.5 µl of 2 × Gflex PCR buffer (Mg^2+^, dNTP plus), 2.0 µl of DNA template, 5.0 µl of 10 µM forward/reverse primers and 0.5 µl of Tks Gflex DNA Polymerase. PCR was performed in “touch down” mode: initial denaturation at 94 °C for 1 min; 10 cycles of 10 s denaturation at 98 °C, 15 s annealing at 69–59 °C (temperature decreased by 1 °C per cycle during the first 10 cycles) and elongation for 30 s/kbp at 68 °C; 28 cycles of 10 s denaturation at 98 °C, 15 s annealing at 59 °C and elongation for 30 s/kbp at 68 °C; final elongation at 68 °C for 5 min. The resulting PCR products were purified by the gel slice method described above and submitted to Eurofin Genomics (Tokyo, Japan) for direct DNA sequencing by Sanger technology. We determined full-length sequences by the primer walking method with designing and synthesizing new specific primers (Online Resource Table S3).

We performed a phylogenetic analysis of the determined nucleotide sequences with the corresponding genomic region of thermophilic hydrogenogenic carboxydotrophs of phylum Firmicutes by the Maximum Likelihood method using MEGA version 7.0.21 (Kumar et al. 2016). Furthermore, ORFs were predicted by using Prodigal version 2.6.3 in the anonymous mode (Hyatt et al. 2010). Genomic synteny was visualized by Easyfig version 2.2.3 with BLASTn (Sullivan et al. 2011). Phylogenetic reconstruction of CODH- and ECH-encoding ORFs was performed as described above.

All nucleotide sequence data obtained in this study are available in the electronic supplementary material (Online Resources Data S1, S2).

## Results

### The optimal CODHech gene identity threshold for species classification

Prior to the development of CODHech-targeted amplicon sequencing, we determined the optimal thresholds for CODHech gene sequence identity for species classification which were used for OTU clustering and classification. We used the genome taxonomic information assigned by GTDB, and the pairwise sequence identities of the 71 full-length CODHech gene sequences to calculate F-measure (Online Resources Table S4; Fig. S1). According to this analysis, pairwise nucleotide sequence identity values between 94.5% and 97.9% garnered the highest F-measure and were considered optimal (Online Resource Fig. S1). Note that the estimated optimal values were higher when we performed the same analysis with the data set limited to the amplification regions of each primer set (Online Resource Fig. S1). This is largely because the intraspecies differences of CODHech gene sequences were rarely observed (i.e. almost all of them were identical) when we used short CODH gene regions. We consider that the optimal thresholds estimated in full-length data set are more appropriate for species classification in our method because too high value (~ 99% in nucleotide identity) might cause overestimation of the diversity and the derivation from uncultured species. Therefore, in this study, we applied the more stringent nucleotide identity value (i.e. 97.9%) for the cutoff of OTU clustering, which is sensitive enough in terms of detection of true biological diversity and robust for PCR errors. Meanwhile, we assigned a CODH-OTU at the species level by using ≥ 94.5% pairwise nucleotide identity with the closest CODHech gene to conservatively estimate the derivation from uncultured species.

### New primer sets for PCR amplification of FirmiCODHech genes

**Fig. 1:**
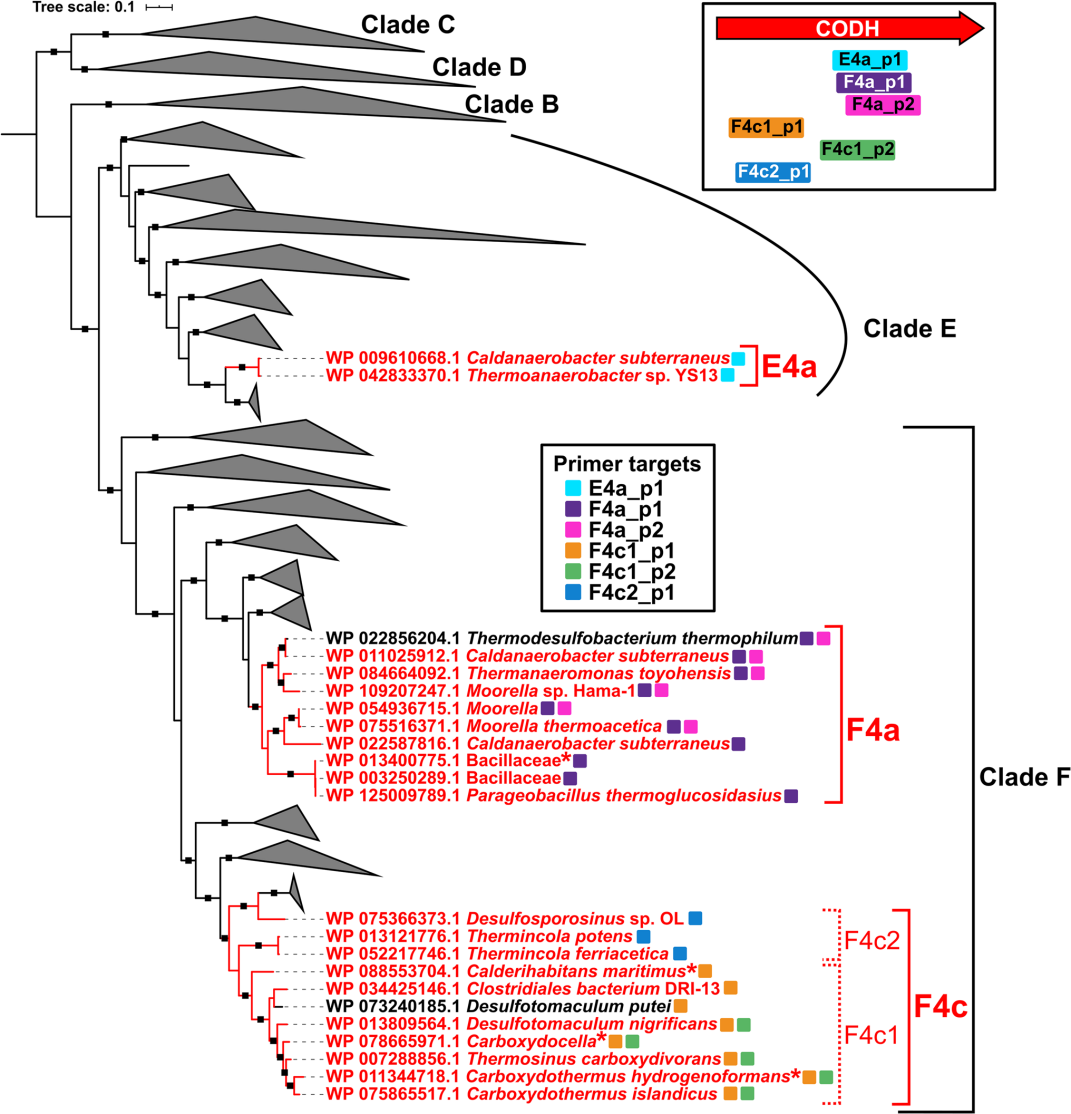
Phylogeny of FirmiCODHech genes and targets of new primers. The tree was constructed using an alignment of 1558 CODH proteins. Major clades B through F are indicated on the right. FirmiCODHech subclades are assigned and colored in red. The colors of the branches and leaves indicate the following: red, FirmiCODHechs; black, other CODHs. The targets of each new primer set are shown by squares placed to the right of the leaves in different colors. Asterisks indicate FirmiCODHech genes added in the CODHech-mock community sample. The black squares on the branch indicate > 0.8 support by bootstrap values. The top right box shows a schematic representation of amplification regions of each primer set

Of the 71 CODHech genes identified in the current genomic database, 34 were derived from members of phylum Firmicutes, a major taxon of thermophilic hydrogenogenic carboxydotrophs with wide distribution (Omae et al. 2019). In this study, we designed new primers targeting these 34 FirmiCODHech genes. According to phylogenetic analysis, the FirmiCODHech genes were found in three different subclades within Clades E and F, which were named E4a, F4a, and F4c according to the phylogenetic clade of the FirmiCODHech and the class of the associated ECH (Group 4 [NiFe] hydrogenase) catalytic subunit (Online Resources Fig. S2; Table S5). While multiple sequence alignment of the 34 FirmiCODHech nucleotide sequences did not identify conserved regions suitable for designing oligonucleotide primers for PCR amplification, conserved regions were found in each subclade (note that F4c had to be split into F4c1 and F4c2) (Online Resource Fig. S3). We newly designed six primer sets for each target group, which collectively covered all 34 FirmiCODHech genes (Table 1; Fig. 1). The F4a_p2 and F4c1_p2 were designed as more specific primer sets with less degeneracy than F4a_p1 and F4c1_p1, respectively. Also note that each primer set was designed at different regions of CODH genes. In particular, while the F4c1_p1 and F4c1_p2 share the target sequences, the amplification regions of them were not overlapped (Fig. 1; Online Resource Fig. S3). Therefore, it is possible that these two primer sets amplify the same gene from the same microorganism, independently.

**Fig. 2:**
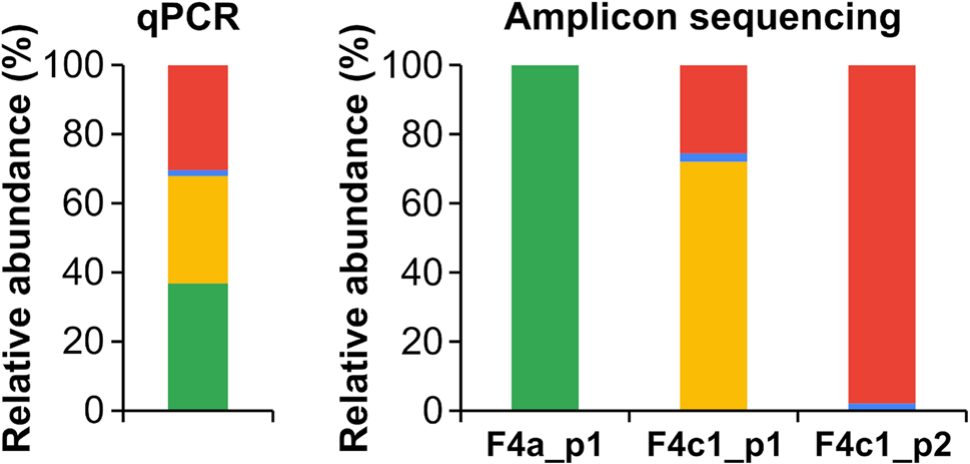
Evaluation of specificity and quantitativity of the new primers using the CODHech-mock community sample. Composition of each FirmiCODHech gene estimated by qPCR is shown on the left. Relative abundance of each CODH-OTU in amplicon sequencing by each new primer set is shown on the right. Each FirmiCODHech gene is shown in a different color: green, AOT13_RS13420 (*Parageobacillus thermoglucosidasius* DSM 2542, F4a); red, CHY_RS08505 (*Carboxydothermus hydrogenoformans* Z-2901, F4c1); blue, ULO1_RS08880 (*Carboxydocella* sp. ULO1, F4c1); yellow, KKC1_RS06675 (*Calderihabitans maritimus* KKC1, F4c1)

The specificity and quantitativity of these primer sets were evaluated by PCR amplification using the CODHech-mock community sample. The DNA extracted from the CODHech-mock community sample contained FirmiCODHech genes of *C hydrogenoformans* Z-2901 (F4c1), *Carboxydocella* sp. ULO1 (F4c1), *C. maritimus* KKC1 (F4c1) and *P. thermoglucosidasius* DSM 2542 (F4a) at 36–760 copies ul^−1^, which were quantified by qPCR using specifically designed primers with 93.7–97.7% efficiency and ≥ 0.998 *R*^2^ values (Fig. 2; Online Resource Table S1). The PCR amplification ability of the three primer sets targeting F4c1 and F4a subclades (F4c1_p1, F4c1_p2 and F4a_p1; Fig. 1) were tested, and the resulting products were of the expected size. Sequencing of the PCR products produced > 64,000 raw paired-end reads per primer set, and the sequence processing left > 28,000 chimera- and noise-free merged reads per primer set (Online Resource Table S6). The CODH-OTUs generated by F4c1_p1, F4c1_p2, and F4a_p1 were identical to the targeting FirmiCODHech genes of each subclade (Fig. 2). Furthermore, the composition of these CODH-OTUs was comparable to the quantification of FirmiCODHech genes by qPCR (Fig. 2). These results collectively indicated that the newly designed primer sets specifically amplified each target and that the amplicon sequencing using them reflects the composition of CODHech genes in the sample. Notably, F4a_p1 generated 42 additional CODH-OTUs, which accounted for ~ 60% of the reads, other than the CODH-OTUs identical to the target FirmiCODHech of *P. thermoglucosidasius* DSM 2542 (Online Resource Table S6). These OTUs were not classified into the F4a subclade, and formed phylogenetically novel clades within Clades E and F (Online Resource Fig. S4), suggesting that these ‘noisy CODH-OTUs’ were derived from unknown species in the sediment sample used for the preparation of the CODHech-mock community sample.

### CODHech genes amplified by new primers from environmental samples

To verify that the newly designed primers amplify CODHech genes from environmental samples, we performed amplicon sequencing using these primers with the sediment samples collected from two hot springs (UN and JI), where thermophilic hydrogenogenic carboxydotrophs have been detected or isolated (Fukuyama et al. 2017; Omae et al. 2019; Yoneda et al. 2012, 2015). PCR amplification was observed for three (F4a_p1, F4c1_p1 and F4c1_p2) and two (F4a_p1 and F4c2_p1) of the six primer sets tested in UN and JI, respectively. Sequencing of the PCR products produced > 68,000 raw paired-end reads per primer per sample, leaving > 39,000 chimera- and noise-free merged reads per primer per sample by sequence processing (Table 2).

**Table 2:** Read statistics of amplicon sequencing in environmental samples

Samples	Primer sets	Total reads	Merged QC reads	Denoised chimera-free reads	CODH-OTUs		CODH-OTUs within FirmiCODHech subclades	
					# of reads	# of OTUs	# of reads	# of OTUs
UN	F4a_p1	81,415	68,345	39,136	37,080	53	0	0
UN	F4c1_p1	68,241	63,494	41,677	41,316	5	41,316	5
UN	F4c1_p2	73,395	67,320	40,855	36,766	3	36,766	3
JI	F4a_p1	112,727	89,115	46,348	41,502	26	0	0
JI	F4c2_p1	169,495	128,748	87,391	62,379	3	62,379	3

**Fig. 3:**
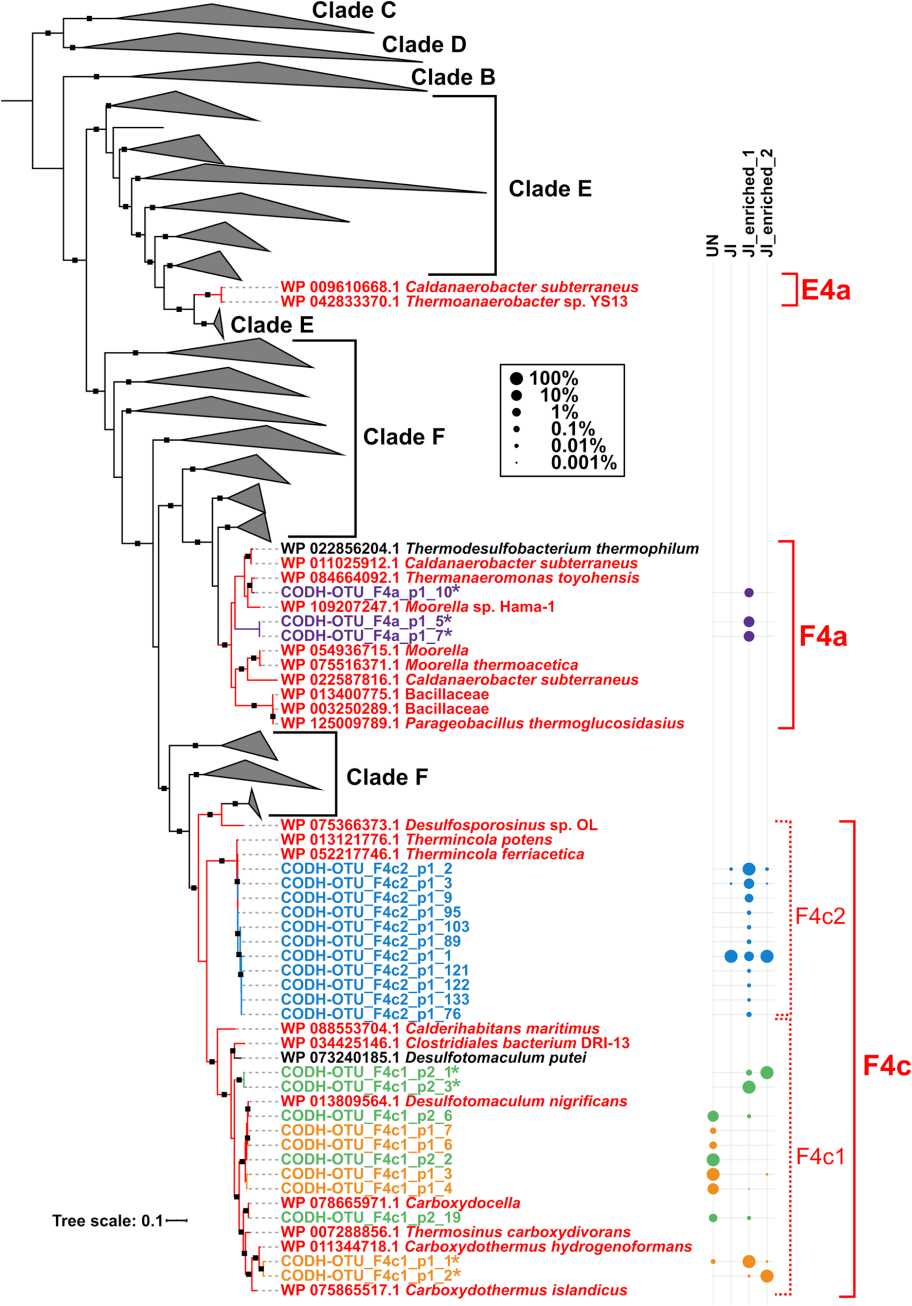
Phylogeny and relative abundance of the CODH-OTUs amplified by the new primer sets from the environmental and enriched samples. The tree was constructed using an alignment of 1558 CODH proteins and the CODH-OTUs. Major clades B through F are indicated on the right. FirmiCODHech subclades are assigned and colored in red. The colors of branches and leaves indicate the following: red, FirmiCODHechs; black, other CODHs; other colors, CODH-OTUs amplified by each new primer set. The relative abundances of CODH-OTUs in each primer set are shown in the bubble plot with different colors to the right of the tree. Asterisks indicate novel CODH-OTUs showing < 94.5% pairwise nucleotide sequence identity with the closest CODHech gene. The black squares on the branch indicate > 0.8 support by bootstrap values

In UN, the CODH reads amplified by the primer sets F4c1_p1 and F4c1_p2 were grouped into five and three CODH-OTUs, respectively, within subclade F4c1 (Table 2; Online Resource Table S7). Most of these CODH-OTUs, which accounted for ~ 99% of read abundance, were phylogenetically related to the CODHech gene of *Desulfotomaculum nigrificans* CO-1-SRB, showing ≥ 98.2% pairwise nucleotide identity (Fig. 3; Online Resource Table S7). CODH-OTUs which were similar or identical to the CODHech genes of *C. hydrogenoformans* Z-2901 and *Carboxydocella* sp. JDF658 were also found, although they accounted for only a small percentage of reads (Fig. 3; Online Resource Table S7). In JI, three CODH-OTUs were generated by F4c2_p1, all of which were determined to be similar to the CODHech genes of *Thermincola* lineages due to their ≥ 97% pairwise nucleotide identity (Fig. 3; Online Resource Table S7). Meanwhile, 53 and 26 CODH-OTUs were generated in UN and JI, respectively, by the primer set F4a_p1 but they were not classified into the F4a FirmiCODHech subclade (Table 2). As is the case in the CODHech-mock community sample, these CODH-OTUs were phylogenetically distinct CODHs forming new clades within Clades E and F, which might be derived from unknown species (Online Resource Fig. S5).

It should be noted that the tree (Fig. 3) was constructed by using full-length alignment containing the regions at which the CODH-OTUs were not aligned (the full-length CODH genes, ~ 650 amino acids; the CODH-OTUs, ~ 150 amino acids) to represent phylogenetic relationships of the CODH-OTUs in the precise reference tree. When we reconstructed the phylogenetic trees using the alignments which were trimmed into each CODH-OTU region, the tree topology was changed from that of full-length tree as expected (Online Resource Fig. S6A). On the other hand, we confirmed that the phylogenetic relationships within each FirmiCODHech subclade were largely conserved even if the trees were reconstructed from the short CODH alignments (Online Resource Fig. S6B). Therefore, we consider that the trimming did not affect our conclusion about the phylogenetic relationships of the CODH-OTUs, and our phylogenetic representation by using the full-length alignment in Fig. 3 does not matter. The same thing can be applicable to the results of the CODH amplicon sequencing with CO-enriched samples, which were mentioned below.

### CODHech genes amplified by the new primer sets from enrichment samples

To verify that the CODH-OTUs identified by the new primers were CODHech genes derived from thermophilic hydrogenogenic carboxydotrophs, we attempted to enrich these microorganisms in the JI sediment sample by incubation under 10% CO. After five days, production of 3.6 and 1.7% of H_2_ was observed in the head spaces of two replicates (JI_enriched_1 and JI_enriched_2, respectively), suggesting that endogenous thermophilic hydrogenogenic carboxydotrophs had actively grown during enrichment. PCR amplification using the new primers elicited products with expected sizes by four and three primer sets in JI_enriched_1 and 2, respectively. Sequencing of the PCR products produced > 71,000 raw paired-end reads per primer per sample, and > 28,000 chimera and noise-free merged reads per primer per sample were left (Table 3).

**Table 3:** Read statistics of amplicon sequencing in CO-enriched samples

Samples	Primer sets	Total reads	Merged QC reads	Denoised chimera-free reads	CODH-OTUs		CODH-OTUs within FirmiCODHech subclades	
					# of reads	# of OTUs	# of reads	# of OTUs
JI_enriched_1	F4a_p1	128,964	108,172	67,696	66,676	21	13,462	3
JI_enriched_1	F4c1_p1	221,536	200,466	138,881	138,881	3	138,881	3
JI_enriched_1	F4c1_p2	92,499	54,465	28,819	11,550	4	11,550	4
JI_enriched_1	F4c2_p1	218,325	207,194	158,881	158,800	12	158,368	11
JI_enriched_2	F4c1_p1	111,569	100,723	63,905	63,905	3	63,905	3
JI_enriched_2	F4c1_p2	71,126	66,414	42,240	42,214	1	42,214	1
JI_enriched_2	F4c2_p1	123,192	116,928	96,397	96,190	4	96,104	3

While only three CODH-OTUs of the F4c2 FirmiCODHech subclade were observed in the original JI sample (Table 2, Online Resource Table S7), we found 22 CODH-OTUs within the F4a, F4c1, and F4c2 subclades in the enriched JI samples (Table 3; Online Resource Table S8). These included 12 CODH-OTUs which were phylogenetically related to the FirmiCODHech genes of known thermophilic hydrogenogenic carboxydotrophs like *Thermincola potens* JR, *D. nigrificans* CO-1-SRB and *Carboxydocella* sp. JDF658 with ≥ 97.7% pairwise nucleotide sequence identities (Byrne-Bailey et al. 2010; Fukuyama et al. 2017; Parshina et al. 2005b; Sokolova et al. 2002; Zavarzina et al. 2007), as well as the three CODH-OTUs of *Thermincola* lineages which were found in the original JI sample (Fig. 3; Online Resource Table S8). While the three *Thermincola* CODH-OTUs found in the original JI sample remained abundant (~ 99% in relative abundance per primer set per sample) in the enriched JI samples, the newly detected 12 CODH-OTUs accounted for a small fraction of the reads obtained (0.001–1% in relative abundance per primer set) (Fig. 3; Online Resource Table S8).

We found seven CODH-OTUs (three in F4a; four in F4c1) which showed < 94.5% pairwise nucleotide sequence identity with the closest FirmiCODHech genes (Fig. 3; Online Resource Table S8). Of the four F4c1 CODH-OTUs, two by primer set F4c1_p2 (F4c1_p2_1 and F4c1_p2_3) formed distinct clades from other known FirmiCODHech genes within the F4c subclade, while the other two by F4c1_p1 (F4c1_p1_1 and F4c1_p1_2) were phylogenetically related to the FirmiCODHech gene of *C. hydrogenoformans* Z-2901 with > 0.8 support by bootstrap (Fig. 3). The three novel F4a CODH-OTUs were amplified only from JI_enriched_1 by primer set F4a_p1, two of which (F4a_p1_5 and F4a_p1_7) were shown to be phylogenetically novel as they formed distinct clades from other known FirmiCODHech genes (Fig. 3). Note that these F4a CODH-OTUs accounted for only ~ 20% of the reads generated by the F4a_p1 primer set; the other reads were grouped into 18 noisy CODH-OTUs, as in the original JI sample (Table 3; Online Resource Fig. S5). The low nucleotide identity scores, which were below the tentative threshold for classifying species in this study, implied that these CODH-OTUs might be derived from uncultured species (Online Resource Fig. S1).

**Fig. 4:**
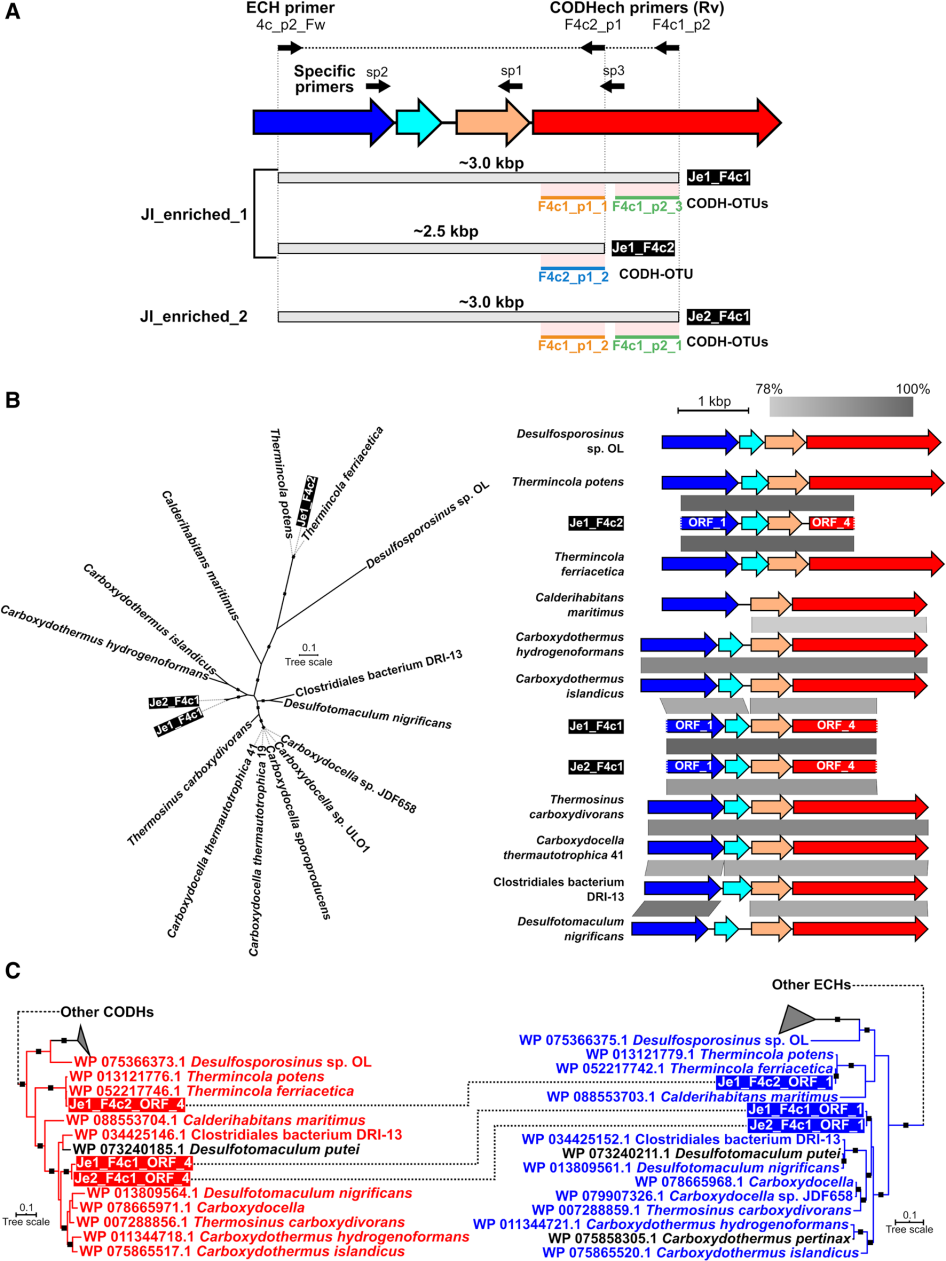
Genomic context and phylogenetic reconstruction of CODH-OTUs found in the enriched samples. **a** Schematic representation of primer regions and determined PCR products. The three determined sequences (Je1_F4c1, Je1_F4a and Je2_F4c1) which contained CODH-OTUs are shown below the gene cluster. **b** ML phylogeny of the nucleotide sequences of the determined sequences (left) and genomic syntenies with their related species (right). For the gene clusters of the Je1_F4c1, Je1_F4a and Je2_F4c1, identifiers of the predicted ORFs are indicated within each gene. **c** Phylogeny of the CODH (left) and the ECH (right) proteins predicted in the determined sequences. Only CODHs of the F4c subclade and FirmiCODHech-associated ECHs (4c class) are shown. In all figures, red and blue colors indicate CODH and ECH genes within the CODH–ECH gene clusters, respectively

To confirm whether the amplified CODH fragments were a part of the CODH–ECH gene clusters, we analyzed the genomic context of the detected CODH-OTUs by direct sequencing with Sanger technology using the new CODH-targeted primer, ECH-targeted primer, and specific primers (Fig. 4a). We successfully amplified and sequenced a ~ 2.5 kbp DNA fragment (Je1_F4c2) with clear peaks from JI_enriched_1 sample (Fig. 4a; Online Resource Table S2). This DNA fragment included the abundant *Thermincola* CODH-OTU (F4c2_p1_2) which accounted for ~ 90% of F4c2_p1 reads (Fig. 3; Online Resource Table S8) and the ~ 2.1 kbp upstream region containing the ECH genes which is similar to the CODH–ECH gene clusters of *Thermincola* isolates (Fig. 4; Online Resource Data S2). In addition, this analysis also identified the genomic contexts of the novel CODH-OTUs (F4c1_p2_1, 3 and F4c1_p1_1, 2) which were abundant (accounting for ~ 100% of the reads per primer per sample) in the CO-enriched samples (Figs. 3, 4; Online Resources Tables S2, S8). Note that these CODH-OTUs which were amplified from non-overlapped regions by F4c1_p1 and F4c1_p2 were sequential: each set of F4c1_p2_3/F4c1_p1_1 and F4c1_p2_1/F4c1_p1_2 were found in each ~ 3.0 kbp DNA fragment (Je1_F4c1 and Je2_F4c1) amplified from JI_enriched_1 and JI_enriched_2, respectively (Fig. 4a; Online Resources Table S2; Data S2). It was revealed that these CODH-OTUs formed gene clusters with ECH genes (Fig. 4b; Online Resource Data S2), and phylogenetic reconstructions based on ECH as well as CODH proteins showed that the Je1_F4c1 and Je2_F4c1 formed distinct clades from other known thermophilic hydrogenogenic carboxydotrophs (Fig. 4c). Therefore, the estimation that the novel CODH-OTUs within F4c1 were derived from the CODHech genes of uncultured hydrogenogenic carboxydotrophs was strongly supported. Note that no PCR amplification was observed when we combined F4a CODH- with associated ECH-targeted primers (F4a_p1_Fw and ECH4a_Rv, respectively), thus we could not reveal their genomic context (Online Resource Table S2). This might be partially explained by the relatively low abundance (~ 10%) of the F4a_p1 CODH-OTUs and difficulty in amplification of long DNA region (Online Resources Tables S2, S8). Overall, it was confirmed that the CODH-OTUs detected in this study were derived from the CODH–ECH gene clusters of hydrogenogenic carboxydotrophs.

## Discussion

In this study, we provided six new primers which could amplify CODHech genes of the phylum Firmicutes and enable to evaluate the diversity of thermophilic hydrogenogenic carboxydotrophs in environmental samples. The new primers were designed to amplify FirmiCODHech genes and applicable to deep-sequencing platform with 300 bp paired-end reads, enabling efficient and high-throughput detection of thermophilic hydrogenogenic carboxydotrophs. Amplicon sequencing with these primers reasonably detected CODH gene fragments which were closely related to the FirmiCODHech genes of known thermophilic hydrogenogenic carboxydotrophs from terrestrial hydrothermal environments. The correct detection of hydrogenogenic carboxydotrophs by these primers was validated not only by the PCR amplification from CO-enriched samples with H_2_ producing activity but also by identification of the ECH genes locating upstream region of the amplified CODH gene fragments by Sanger technology. In addition, our method successfully identified phylogenetically novel CODHech genes which might be derived from uncultured hydrogenogenic carboxydotrophs. Correctively, our primers are good molecular ecological tools for diversity estimation of these microorganisms.

To our knowledge, only one study has previously designed degenerate primers to amplify CODH genes for diversity analysis (Matson et al. 2011). In contrast to the new primers provided here, these previous primers were designed to match broad sequences which correspond to 27% of CODH genes in microbial genomes. In addition, unlike the new primers, the amplicon size of the previous primers is ~ 1300 bp (Matson et al. 2011), which is not applicable to short-read deep-sequencing platform. Therefore, for the purpose of diversity analysis of thermophilic hydrogenogenic carboxydotrophs, the new primers are more appropriate than the previous ones. In fact, although CODH genes associated with ACS or CooF in Clades C and E have been amplified by the previous primers, CODHech genes have never been amplified from environments (Hoshino and Inagaki 2017; Matson et al. 2011). Therefore, our study is the first to estimate CODH-based diversity of hydrogenogenic carboxydotrophs.

Although most primer sets specifically amplified their target-CODHech genes, only the primer set F4a_p1 yielded noisy CODH-OTUs within Clades E and F (Online Resources Figs. S4, S5). Clades E and F are large and diverse groups of CODH genes: they include CODH genes of CODH–ACS, CODH–CooF, CODH–ECH and some other gene clusters from (hyper-)thermophilic and mesophilic archaea and bacteria (Inoue et al. 2019; Techtmann et al. 2012). Therefore, we could not identify the function of these noisy CODH-OTUs which might be derived from uncultured species. In future, we should revise this primer to be more specific by using a larger CODH database, including the phylogenetically novel CODH genes. Meanwhile, the F4a_p1 amplified the F4a FirmiCODHech genes from the enriched sample (Fig. 3) and seems to be applicable to diversity analysis of thermophilic hydrogenogenic carboxydotrophs.

We showed that the pairwise nucleotide sequence identities of 94.5–97.9% were optimal for species classification (Online Resource Fig S1). These values seems relatively high, compared to those ranging 85–90% in other amplicon sequencing studies targeting cytochrome-*cd*_1_ nitrite reductase (Lee and Francis 2017), dissimilatory sulfite reductase (Pelikan et al. 2016) and ammonia monooxygenase (Pester et al. 2012). This might imply intraspecies conservation of CODHech genes within Firmicutes, while the CODHech genes are often horizontally transferred among different or same species (Techtmann et al. 2012; Sant’Anna et al. 2015). In our analysis, the more stringent nucleotide identity value (97.9%) was applied for the cutoff of OTU clustering to distinguish true biological variation. We consider that the gene diversity was not overestimated, because CODH-OTUs which were clustered by this threshold correctly predicted the presence of highly similar CODHech genes which were identified by resequencing by Sanger technology (Je1_F4c1_ORF_4 and Je2_F4c1_ORF_4; 97.8% pairwise nucleotide identity) (Fig. 4; Online Resource Data S2). These results suggested that our method provides a fine-grained analysis of thermophilic hydrogenogenic carboxydotrophs, which reveals their diversity at species or strain level.

Some CODH-OTUs including those on Je1_F4c1 and Je2_F4c1 showed < 94.5% pairwise nucleotide sequence identity with the closest FirmiCODHechs. These CODH-OTUs conserved the His and Cys residues in the C-cluster of CODH genes, which is a catalytic metal cluster comprising Ni, Fe, and S (Dobbek et al. 2001; Inoue et al. 2019), suggesting that these genes possess a conserved CODH function. Furthermore, we successfully revealed that the CODH-OTUs and the associated ECH genes formed phylogenetically distinct subclades (Fig. 4c) while they formed sister clades with genes of typical thermophilic bacterial hydrogenogenic carboxydotrophs like *C. hydrogenoformans* (Wu et al. 2005), *Carboxydocella* spcecies (Fukuyama et al. 2017; Toshchakov et al. 2018), *D. nigrificans* CO-1-SRB (Parshina et al. 2005b) and *Thermosinus carboxydivorans* (Sokolova et al. 2004) and their genomic contexts were also well conserved (Fig. 4b). Therefore, our new primers enabled us to evaluate unknown thermophilic hydrogenogenic carboxydotrophs without isolation. Meanwhile, it should be noted that the taxonomic assignment of these phylogenetically distinct CODH-OTUs is difficult because CODHech genes are considered to be often horizontally transferred and taxonomically remote species are found in FirmiCODHech subclades. Further studies like targeted metagenomics (Grieb et al. 2020) or isolation (Batani et al. 2019; Lewis et al. 2020) methods which have been recently developed are needed to obtain more genomic information for taxonomic assignment of these CODH-OTUs.

Most of the CODH-OTUs found in CO-enriched samples were not detected in the JI sample before enrichment (Fig. 3). These results suggested that the abundance of thermophilic hydrogenogenic carboxydotrophs before enrichment was smaller than the detection limit. This observation corresponds to previous studies (Brady et al. 2015; Omae et al. 2019; Yoneda et al. 2015) and suggests that these microorganisms might compose a rare biosphere and show microbial seed bank dynamics in the environment (Lynch and Neufeld 2015). Metagenomic survey rarely founds ECH genes (Greening et al. 2016) , also suggesting that these microorganisms might occur at the low relative abundance in most environments. It is predicted that unexplored rare thermophilic hydrogenogenic carboxydotrophs exist in a wide variety of environments and CODH amplicon sequencing in combination with CO-enrichment is one effective option for unveiling these microorganisms in high-resolution.

Overall, we provided high-throughput deep-sequencing screening of thermophilic hydrogenogenic carboxydotrophs based on CODH genes. Further studies using these primers in a wide variety of environments, including lake or marine sediment, soil, and compost will reveal the diversity and distribution of thermophilic hydrogenogenic carboxydotrophs, including that of unknown species.

## Supplementary Information

Below is the link to the electronic supplementary material.

Supplementary file1 (PDF 2358 KB)
