# Supplementary material for: Diversity analysis of thermophilic hydrogenogenic carboxydotrophs by carbon monoxide dehydrogenase amplicon sequencing using new primers
Source: Extremophiles. 2021 Jan 7;25(1):61–76. doi: 10.1007/s00792-020-01211-y (PMC7811984; doi:10.1007/s00792-020-01211-y)
Supplement: Supplementary file 1 — Supplementary file1 (PDF 2358 KB) [file 792_2020_1211_MOESM1_ESM.pdf]

Electronic Supplementary Material

Journal: Extremophiles

Title:
Diversity analysis of thermophilic hydrogenogenic carboxydotrophs by carbon monoxide dehydrogenase amplicon sequencing using new primers

Kimiho Omae, Tatsuki Oguro, Masao Inoue, Yuto Fukuyama, Takashi Yoshida and Yoshihiko Sako

Correspondence:
Takashi Yoshida, Laboratory of Marine Microbiology, Graduate School of Agriculture, Kyoto University, Kitashirakawa Oiwake-cho, Sakyo-ku, Kyoto 606-8503, Japan.
E-mail: yoshiten@kais.kyoto-u.ac.jp

Table S1 Primer sequences and efficiency of amplification for qPCR

Primer target	Fw primer sequence (5' -> 3')	Rv primer sequence (5' -> 3')	Amplification efficieancy (%)	R^2
CHY_RS08505 (Carboxydothermus hydrogenoformans Z-2901)	GCTAATGACCCGTTAAAACC	GTTACAACCGGCAAATAAGG	93.7	0.999
ULO1_RS08880 (*Carboxydocella* sp. ULO1)	TAACAATCCTAAGGCGATCC	TGCCTCCTGAGTCATTAAAC	96.4	0.998
KKC1_RS06675 (*Calderihabitans maritimus* KKC1)	TGTCGCATTTGTTGGAAGGG	ATGGTATCGGCATCAGCACC	93.9	0.999
AOT13_RS13420 (*Parageobacillus thermoglucosidasius* DSM 2542)	TCCGCGGAGTCTGTCTCTTTG	CAACCAGTAGCCAGCAGCAG	97.7	0.999

Table S2 The results of PCR amplification of genetic regions between CODH-OTUs and associated ECH genes

DNA template	Fw primer	Rv primer	Amplification	DNA fragment name	Product size
JI_enriched_1	ECH4c_Fw	CODH_F4c1_p2_Rv	Yes	F4c1_JI_enriched_1	~3 kbp
JI_enriched_1	ECH4c_Fw	CODH_F4c2_p1_Rv	Yes	F4c2_JI_enriched_1	~2.5 kbp
JI_enriched_2	ECH4c_Fw	CODH_F4c1_p2_Rv	Yes	F4c1_JI_enriched_2	~3 kbp
JI_enriched_1	CODH_F4a_p1_Fw	ECH4a_Rv	No	-	~9 kbp*

* Expected product size is shown.

Table S3 Specific primer sequences used to determine the genomic context of CODH-OTUs

Name	Sequence (5' -> 3')
sp1_F4c1_Rv	CTCTCGTAATCTACCAGCATTATAGC
sp2_F4c1_Fw	GAAGCGCTTCATGTTATGATACCGG
sp3_F4c1_Rv	GGTTCAGGGGTACCAAATAAGGC
sp1_F4c2_Rv	GATCAACCAGCATTACAGCCTGC
sp2_F4c2_Fw	CTGGGGAGGCAGTTGCCAAG

Table S4 GTDB taxonomy of genomes harboring CODHech genes

Accession	NCBI taxonomy	GTDB taxonomy
GCF_003947435.1	*Candidatus* Methanodesulfokores washburnensis	*Candidatus* Methanodesulfokores washburnensis[a]
GCF_000813245.1	*Thermofilum carboxyditrophus* 1505	*Thermofilum carboxyditrophus*
GCF_000816105.1	*Thermococcus guaymasensis* DSM 11113	*Thermococcus guaymasensis*
GCF_000018365.1	*Thermococcus onnurineus* NA1	*Thermococcus onnurineus*
GCF_000151205.2	*Thermococcus* sp. AM4	*Thermococcus* sp000151205
GCF_000151105.2	*Thermococcus barophilus* MP	*Thermococcus_B barophilus*
GCF_001433455.1	*Thermococcus barophilus* CH5	*Thermococcus_B barophilus*
GCF_000517445.1	*Thermococcus paralvinellae* ES1	*Thermococcus_B paralvinellae*
GCF_000166075.1	*Parageobacillus thermoglucosidasius* Y4.1MC1	*Parageobacillus thermoglucosidasius*
GCF_000178395.2	*Parageobacillus thermoglucosidasius* C56-YS93	*Parageobacillus thermoglucosidasius*
GCF_000258725.1	*Parageobacillus thermoglucosidasius* TNO-09.020	*Parageobacillus thermoglucosidasius*
GCF_000648295.1	*Parageobacillus thermoglucosidasius* NBRC 107763	*Parageobacillus thermoglucosidasius*
GCF_000966225.1	*Parageobacillus thermoglucosidasius* DSM 2542	*Parageobacillus thermoglucosidasius*
GCF_001295365.1	*Parageobacillus thermoglucosidasius* DSM 2542	*Parageobacillus thermoglucosidasius*
GCF_001587555.1	*Parageobacillus thermoglucosidasius* B4168	*Parageobacillus thermoglucosidasius*
GCF_001651535.1	*Parageobacillus thermoglucosidasius* GT23	*Parageobacillus thermoglucosidasius*
GCF_001700985.1	*Parageobacillus thermoglucosidasius* NCIMB 11955	*Parageobacillus thermoglucosidasius*
GCF_001902495.1	*Parageobacillus thermoglucosidasius* TM242	*Parageobacillus thermoglucosidasius*
GCF_003865195.1	*Parageobacillus thermoglucosidasius* TG4	*Parageobacillus thermoglucosidasius*
GCF_000156275.2	*Caldanaerobacter subterraneus* subsp. *pacificus* DSM 12653	*Caldanaerobacter subterraneus*
GCF_000007085.1	*Caldanaerobacter subterraneus* subsp. *tengcongensis* MB4	*Caldanaerobacter subterraneus*
GCF_000473865.1	*Caldanaerobacter subterraneus* subsp. *yonseiensis* KB-1	*Caldanaerobacter subterraneus*
GCF_000806225.2	*Thermoanaerobacter* sp. YS13	*Thermoanaerobacter uzonensis*
GCF_001936615.1	*Desulfosporosinus* sp. OL	*Desulfosporosinus* sp001936615
GCF_000012865.1	*Carboxydothermus hydrogenoformans* Z-2901	*Carboxydothermus hydrogenoformans*
GCF_001950325.1	*Carboxydothermus islandicus* SET	*Carboxydothermus islandicus*

GCF_000214435.1	*Desulfotomaculum nigrificans* CO-1-SRB	*Desulfotomaculum nigrificans*
GCF_002207765.1	*Calderihabitans maritimus* KKC1	*Calderihabitans maritimus*
GCF_003116935.1	*Moorella* sp. Hama-1	*Moorella* sp003116935
GCF_001373375.1	*Moorella glycerini* NMP	*Moorella stamsii*
GCF_002995805.1	*Moorella stamsii* DSM 26271	*Moorella stamsii*
GCF_001875325.1	*Moorella thermoacetica* DSM 21394	*Moorella thermoacetica_A*
GCF_900176005.1	*Thermanaeromonas toyohensis* ToBE	*Thermanaeromonas toyohensis*
GCF_000746025.1	Clostridiales bacterium DRI-13	DRI-13 sp000746025
GCF_000092945.1	*Thermincola potens* JR	*Thermincola ferriacetica*
GCF_001263415.1	*Thermincola ferriacetica* Z-0001	*Thermincola ferriacetica*
GCF_002049255.1	*Carboxydocella* sp. ULO1	*Carboxydocella thermautotrophica*
GCF_002049395.1	*Carboxydocella* sp. JDF658	*Carboxydocella thermautotrophica*
GCF_003047205.1	*Carboxydocella thermautotrophica* 19	*Carboxydocella thermautotrophica*
GCF_003054495.1	*Carboxydocella thermautotrophica* 41	*Carboxydocella thermautotrophica*
GCF_900167165.1	*Carboxydocella sporoproducens* DSM 16521	*Carboxydocella thermautotrophica*
GCF_000169155.1	*Thermosinus carboxydivorans* Nor1	*Thermosinus carboxydivorans*
GCF_900167065.1	*Desulfovibrio bizertensis* DSM 18034	*Desulfovibrio_O bizertensis*
GCF_000341895.1	*Pseudodesulfovibrio piezophilus* C1TLV30	*Pseudodesulfovibrio piezophilus*
GCF_000817955.1	*Geobacter pickeringii* G13	*Geobacter pickeringii*
GCF_000020725.1	*Geobacter bemidjiensis* Bem	*Geobacter_A bemidjiensis*
GCF_002770725.1	*Pleomorphomonas carboxyditropha* SVCO-16	*Pleomorphomonas* sp900095415
GCF_900466875.1	Rhizobiales bacterium AFS016371	*Neorhizobium* unculture_A[b]
GCF_900468955.1	Rhizobiales bacterium AFS041951	*Neorhizobium* unculture_B[b]
GCF_900469445.1	Rhizobiales bacterium AFS049984	*Neorhizobium* unculture_B[b]
GCF_900472805.1	Rhizobiales bacterium AFS089140	*Neorhizobium* unculture_B[b]
GCF_900104145.1	*Pseudovibrio* sp. Tun.PSC04-5.I4	*Pseudovibrio* sp900104145
GCF_001431305.1	*Pseudovibrio* sp. POLY-S9	*Pseudovibrio* sp900143565
GCF_000013745.1	*Rhodopseudomonas palustris* BisB18	*Rhodopseudomonas palustris_D*

GCF_000013085.1	*Rhodospirillum rubrum* ATCC 11170	*Rhodospirillum rubrum*
GCF_000225955.1	*Rhodospirillum rubrum* F11	*Rhodospirillum rubrum*
GCF_000981805.1	*Citrobacter amalonaticus* Y19	*Citrobacter_A amalonaticus_C*
GCF_000263295.1	*Salmonella enterica* subsp. *enterica* serovar Senftenberg SS209	*Salmonella enterica*
GCF_001276695.1	*Salmonella enterica* subsp. *enterica* serovar Montevideo 50262	*Salmonella enterica*
GCF_001276745.1	*Salmonella enterica* subsp. *enterica* serovar Senftenberg 50263	*Salmonella enterica*
GCF_001276765.1	*Salmonella enterica* subsp. *enterica* serovar Senftenberg 50264	*Salmonella enterica*
GCF_001276775.1	*Salmonella enterica* subsp. *enterica* serovar Senftenberg 50265	*Salmonella enterica*
GCF_001276825.1	*Salmonella enterica* subsp. *enterica* serovar Senftenberg 50271	*Salmonella enterica*
GCF_001276905.1	*Salmonella enterica* subsp. *enterica* serovar Montevideo 50270	*Salmonella enterica*
GCF_001276925.1	*Salmonella enterica* subsp. *enterica* serovar Senftenberg 50272	*Salmonella enterica*
GCF_000422645.1	*Ferrimonas futtsuensis* DSM 18154	*Ferrimonas futtsuensis*
GCF_000425405.1	*Ferrimonas kyonanensis* DSM 18153	*Ferrimonas kyonanensis*
GCF_900100175.1	*Ferrimonas sediminum* DSM 23317	*Ferrimonas sediminum*
GCF_003797165.1	*Shewanella* sp. R106	GCF_002836275.1
GCF_003855155.1	*Shewanella* sp. M2	GCF_002836275.1
GCF_000331515.1	*Photobacterium marinum* AK15	*Photobacterium marinum*

[a] NCBI taxonomy was used, because GTDB taxonomy was not assigned.

[b] Species-level taxonomy was manually assigned by calculating ANI.

Table S5 Genomes harboring FirmiCODHech and associated ECH (Group 4 [NiFe] hydrogenase)

**Genome**		**FirmiCODHech**			**Group 4 [NiFe] hydrogenase catalytic subunit**		
**Accession**	**Taxonomy**	**Accession**	**Locus tag**	**Sub-clade**	**Accession**	**Locus tag**	**Class**
GCF_000156275.2	*Caldanaerobacter subterraneus* subsp. *pacificus* DSM 12653	WP_009610668.1	CDSM653_RS07355	E4a	WP_009610669.1	CDSM653_RS07320	4a
GCF_000806225.2	*Thermoanaerobacter* sp. YS13	WP_042833370.1	THYS13_RS04710	E4a	WP_042833365.1	THYS13_RS04650	4a
GCF_000166075.1	*Parageobacillus thermoglucosidasius* Y4.1MC1	WP_013400775.1	GY4MC1_RS09120	F4a	WP_013400781.1	GY4MC1_RS09155	4a
GCF_000178395.2	*Parageobacillus thermoglucosidasius* C56-YS93	WP_013400775.1	GEOTH_RS09250	F4a	WP_013876883.1	GEOTH_RS09285	4a
GCF_000258725.1	*Parageobacillus thermoglucosidasius* TNO-09.020	WP_003250289.1	GT20_RS08580	F4a	WP_003250304.1	GT20_RS08615	4a
GCF_000648295.1	*Parageobacillus thermoglucosidasius* NBRC 107763	WP_013400775.1	GT2_RS13950	F4a	WP_013400781.1	GT2_RS13985	4a
GCF_000966225.1	*Parageobacillus thermoglucosidasius* DSM 2542	WP_013400775.1	WH82_RS08365	F4a	WP_013400781.1	WH82_RS08330	4a
GCF_001295365.1	*Parageobacillus thermoglucosidasius* DSM 2542	WP_013400775.1	AOT13_RS13420	F4a	WP_013400781.1	AOT13_RS13385	4a
GCF_001587555.1	*Parageobacillus thermoglucosidasius* B4168	WP_003250289.1	B4168_RS01110	F4a	WP_003250304.1	B4168_RS01145	4a
GCF_001651535.1	*Parageobacillus thermoglucosidasius* GT23	WP_003250289.1	GT23_RS00370	F4a	WP_003250304.1	GT23_RS00335	4a
GCF_001700985.1	*Parageobacillus thermoglucosidasius* NCIMB 11955	WP_013400775.1	BCV53_RS13415	F4a	WP_013400781.1	BCV53_RS13380	4a
GCF_001902495.1	*Parageobacillus thermoglucosidasius* TM242	WP_013400775.1	BCV54_RS13805	F4a	WP_013400781.1	BCV54_RS13770	4a
GCF_003865195.1	*Parageobacillus thermoglucosidasius* TG4	WP_125009789.1	PTHTG4_RS08120	F4a	WP_125009791.1	PTHTG4_RS08155	4a
GCF_000007085.1	*Caldanaerobacter subterraneus* subsp. *tengcongensis* MB4	WP_011025912.1	TTE_RS08175	F4a	WP_009610669.1	TTE_RS08140	4a
GCF_000473865.1	*Caldanaerobacter subterraneus* subsp. *yonseiensis* KB-1	WP_022587816.1	O163_RS22180	F4a	WP_022587809.1	O163_RS22145	4a
GCF_003116935.1	*Moorella* sp. Hama-1	WP_109207247.1	Hama1_RS10675	F4a	WP_109207254.1	Hama1_RS10710	4a
GCF_001373375.1	*Moorella glycerini* NMP	WP_054936715.1	BN1230_RS08005	F4a	WP_054936708.1	BN1230_RS07970	4a
GCF_002995805.1	*Moorella stamsii* DSM 26271	WP_054936715.1	MOST_RS16220	F4a	WP_054936708.1	MOST_RS16185	4a
GCF_001875325.1	*Moorella thermoacetica* DSM 21394	WP_075516371.1	MOTE_RS04415	F4a	WP_075516364.1	MOTE_RS04380	4a
GCF_900176005.1	*Thermanaeromonas toyohensis* ToBE	WP_084664092.1	B9A14_RS03610	F4a	WP_084664078.1	B9A14_RS03575	4a
GCF_000012865.1	*Carboxydothermus hydrogenoformans* Z-2901	WP_011344718.1	CHY_RS08505	F4c1	WP_011344721.1	CHY_RS08520	4c
GCF_001950325.1	*Carboxydothermus islandicus* SET	WP_075865517.1	ciss_RS06710	F4c1	WP_075865520.1	ciss_RS06725	4c
GCF_000214435.1	*Desulfotomaculum nigrificans* CO-1-SRB	WP_013809564.1	DESCA_RS01885	F4c1	WP_013809561.1	DESCA_RS01870	4c
GCF_002207765.1	*Calderihabitans maritimus* KKC1	WP_088553704.1	KKC1_RS06675	F4c1	WP_088553703.1	KKC1_RS06665	4c
GCF_000746025.1	Clostridiales bacterium DRI-13	WP_034425146.1	BR63_RS15745	F4c1	WP_034425152.1	BR63_RS15760	4c

GCF_002049255.1	*Carboxydocella* sp. ULO1	WP_078665971.1	ULO1_RS08880	F4c1	WP_078665968.1	ULO1_RS08895	4c
GCF_002049395.1	*Carboxydocella* sp. JDF658	WP_078665971.1	JDF658_RS09120	F4c1	WP_079907326.1	JDF658_RS09135	4c
GCF_003047205.1	*Carboxydocella thermautotrophica* 19	WP_078665971.1	CFE_RS00860	F4c1	WP_078665968.1	CFE_RS00845	4c
GCF_003054495.1	*Carboxydocella thermautotrophica* 41	WP_078665971.1	CTH_RS00865	F4c1	WP_078665968.1	CTH_RS00850	4c
GCF_900167165.1	*Carboxydocella sporoproducens* DSM 16521	WP_078665971.1	B5D20_RS09335	F4c1	WP_078665968.1	B5D20_RS09320	4c
GCF_000169155.1	*Thermosinus carboxydivorans* Nor1	WP_007288856.1	TCARDRAFT_RS04635	F4c1	WP_007288859.1	TCARDRAFT_RS04650	4c
GCF_001936615.1	*Desulfosporosinus* sp. OL	WP_075366373.1	DSOL_RS18930	F4c2	WP_075366375.1	DSOL_RS18945	4c
GCF_000092945.1	*Thermincola potens* JR	WP_013121776.1	THERJR_RS14890	F4c2	WP_013121779.1	THERJR_RS14905	4c
GCF_001263415.1	*Thermincola ferriacetica* Z-0001	WP_052217746.1	Tfer_RS07525	F4c2	WP_052217742.1	Tfer_RS07510	4c

Table S6 Read statistics of amplicon sequencing in the CODHech-mock community sample

**Primer sets**	**Total reads**	**Merged QC reads**	**Denoised chimera-free reads**	**CODH reads**	**CODH-OTUs**
F4a_p1	122,285	88,442	42,364	42,354	43
F4c1_p1	65,790	57,720	30,129	30,129	3
F4c1_p2	64,059	54,321	28,610	28,610	2

Table S7 Environmental CODH-OTUs classified into FirmiCODHech subclades

CODH-OTUs*	Sub-clades	Closest CODHech genes			Relative abundance in each primer set (%)	
		**Locus tag**	**Taxon**	**Identity (%)**	**UN**	**JI**
**F4c1_p1_1**	F4c1	CHY_RS08505	*Carboxydothermus hydrogenoformans* Z-2901	**93.8**	0.017	0
F4c1_p1_3	F4c1	DESCA_RS01885	*Desulfotomaculum nigrificans* CO-1-SRB	98.2	85.599	0
F4c1_p1_4	F4c1	DESCA_RS01885	*Desulfotomaculum nigrificans* CO-1-SRB	98.7	13.975	0
F4c1_p1_6	F4c1	DESCA_RS01885	*Desulfotomaculum nigrificans* CO-1-SRB	99.1	0.327	0
F4c1_p1_7	F4c1	DESCA_RS01885	*Desulfotomaculum nigrificans* CO-1-SRB	99.3	0.082	0
F4c1_p2_2	F4c1	DESCA_RS01885	*Desulfotomaculum nigrificans* CO-1-SRB	99.1	85.566	0
F4c1_p2_6	F4c1	DESCA_RS01885	*Desulfotomaculum nigrificans* CO-1-SRB	99.8	13.624	0
F4c1_p2_19	F4c1	JDF658_RS09120	*Carboxydocella* sp. JDF658	100	0.811	0
F4c2_p1_1	F4c2	THERJR_RS14890	*Thermincola potens* JR	97	0	99.994
F4c2_p1_2	F4c2	Tfer_RS07525	*Thermincola ferriacetica* Z-0001	99.8	0	0.005
F4c2_p1_3	F4c2	Tfer_RS07525	*Thermincola ferriacetica* Z-0001	99.3	0	0.002

* CODH-OTU names were represented in the name of primer set which amplified it, and number identifier of the CODH-OTU, which were concatenated with "_" characters. CODH-OTUs which showed <94.5% identity with the closest CODHech genes were shown in bold.

Table S8 CODH-OTUs in CO-enriched samples classified into FirmiCODHech subclades

CODH-OTUs*	Sub-clades	Closest CODHech genes			Relative abundance in each primer set (%)	
		Locus tag	Taxon	Identity (%)	JI_enriched_1	JI_enriched_2
**F4a_p1_5**	F4a	Hama1_RS10675	*Moorella* sp. Hama 1	**85.7**	9.854	0
**F4a_p1_7**	F4a	Hama1_RS10675	*Moorella* sp. Hama 1	**85.7**	8.355	0
**F4a_p1_10**	F4a	B9A14_RS03610	*Thermanaeromonas toyohensis* ToBE	**92.9**	1.981	0
**F4c1_p1_1**	F4c1	CHY_RS08505	*Carboxydothermus hydrogenoformans* Z-2901	**93.8**	99.997	0.002
**F4c1_p1_2**	F4c1	CHY_RS08505	*Carboxydothermus hydrogenoformans* Z-2901	**93.4**	0.002	99.997
F4c1_p1_3	F4c1	DESCA_RS01885	*Desulfotomaculum nigrificans* CO-1-SRB	98.2	0	0.002
F4c1_p1_4	F4c1	DESCA_RS01885	*Desulfotomaculum nigrificans* CO-1-SRB	98.7	0.001	0
**F4c1_p2_1**	F4c1	DESCA_RS01885	*Desulfotomaculum nigrificans* CO-1-SRB	**89.2**	0.061	100
**F4c1_p2_3**	F4c1	DESCA_RS01885	*Desulfotomaculum nigrificans* CO-1-SRB	**88.5**	99.922	0
F4c1_p2_6	F4c1	DESCA_RS01885	*Desulfotomaculum nigrificans* CO-1-SRB	99.8	0.009	0
F4c1_p2_19	F4c1	JDF658_RS09120	*Carboxydocella* sp. JDF658	100	0.009	0
F4c2_p1_1	F4c2	THERJR_RS14890	*Thermincola potens* JR	97	2.903	99.904
F4c2_p1_2	F4c2	Tfer_RS07525	*Thermincola ferriacetica* Z-0001	99.8	89.416	0.005
F4c2_p1_3	F4c2	Tfer_RS07525	*Thermincola ferriacetica* Z-0001	99.3	6.3	0.001
F4c2_p1_9	F4c2	Tfer_RS07525	*Thermincola ferriacetica* Z-0001	99.1	1.025	0
F4c2_p1_76	F4c2	THERJR_RS14890	*Thermincola potens* JR	98.1	0.027	0
F4c2_p1_89	F4c2	Tfer_RS07525	*Thermincola ferriacetica* Z-0001	98.4	0.015	0
F4c2_p1_95	F4c2	Tfer_RS07525	*Thermincola ferriacetica* Z-0001	98.4	0.012	0
F4c2_p1_103	F4c2	THERJR_RS14890	*Thermincola potens* JR	98.4	0.011	0
F4c2_p1_121	F4c2	THERJR_RS14890	*Thermincola potens* JR	97.7	0.008	0
F4c2_p1_122	F4c2	THERJR_RS14890	*Thermincola potens* JR	98.1	0.008	0
F4c2_p1_133	F4c2	THERJR_RS14890	*Thermincola potens* JR	97.9	0.005	0

* CODH-OTU names were represented in the name of primer set which amplified it, and number identifier of the CODH-OTU, which were concatenated with "_" characters. CODH-OTUs which showed <94.5% identity with the closest CODHech genes were shown in bold.

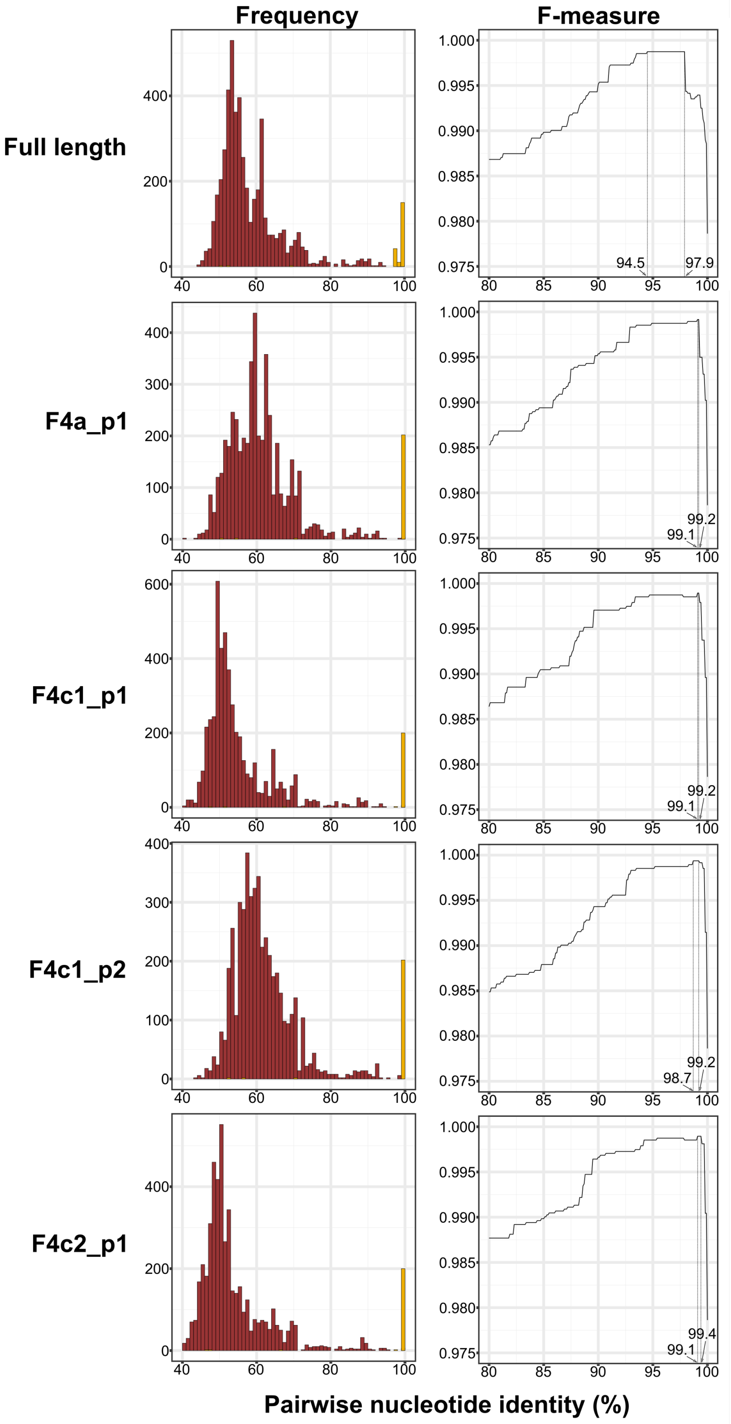

Fig. S1 Optimal thresholds for species classifications of CODHech genes. Pairwise sequence identity distribution (left) and F-measure (right) based on all CODHech genes. Each color represents the pairwise nucleotide identity calculations between two strains belonging to the same species (yellow) and belonging to different species (red) in left columns. Each row shows the result of the same analysis using different data set without trimming (full length) or with trimming into each primer set region (others).

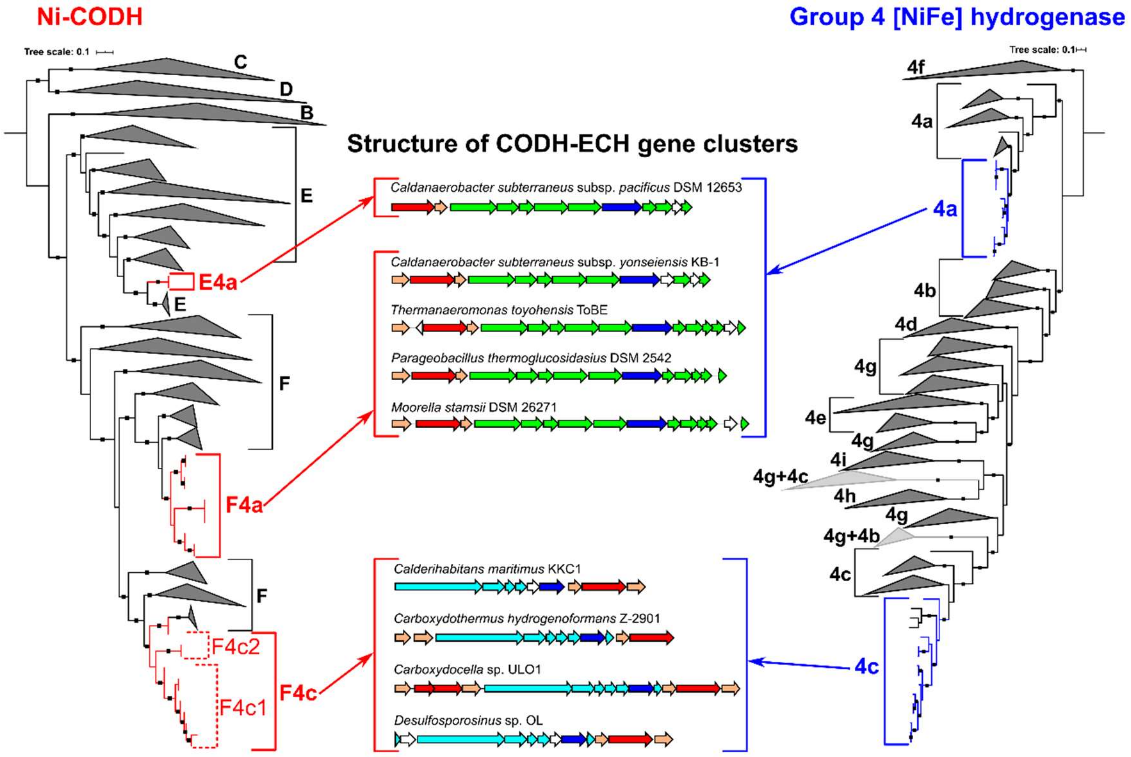

Fig. S2 Structural and phylogenetic classification of CODH–ECH gene clusters. Phylogenetic trees of CODH genes and Group 4 [NiFe] hydrogenase catalytic subunit genes are shown on the left and right, respectively. The names of major clades are shown in black characters and branches in the trees based on previous studies (Inoue *et al*. 2019b; Søndergaard *et al*. 2016; Techtmann *et al*. 2012). Black squares indicate >0.8 support by bootstrap values. Structures of representative CODH–ECH gene clusters are shown at the center. Red = CODH and, and blue = Group 4 [NiFe] hydrogenase catalytic subunit genes within the CODH–ECH gene clusters. Orange arrows indicate CODH-related genes (*cooF* or *cooC*). Light green and light blue arrows indicate Group 4a and 4c [NiFe] hydrogenase related genes, respectively.

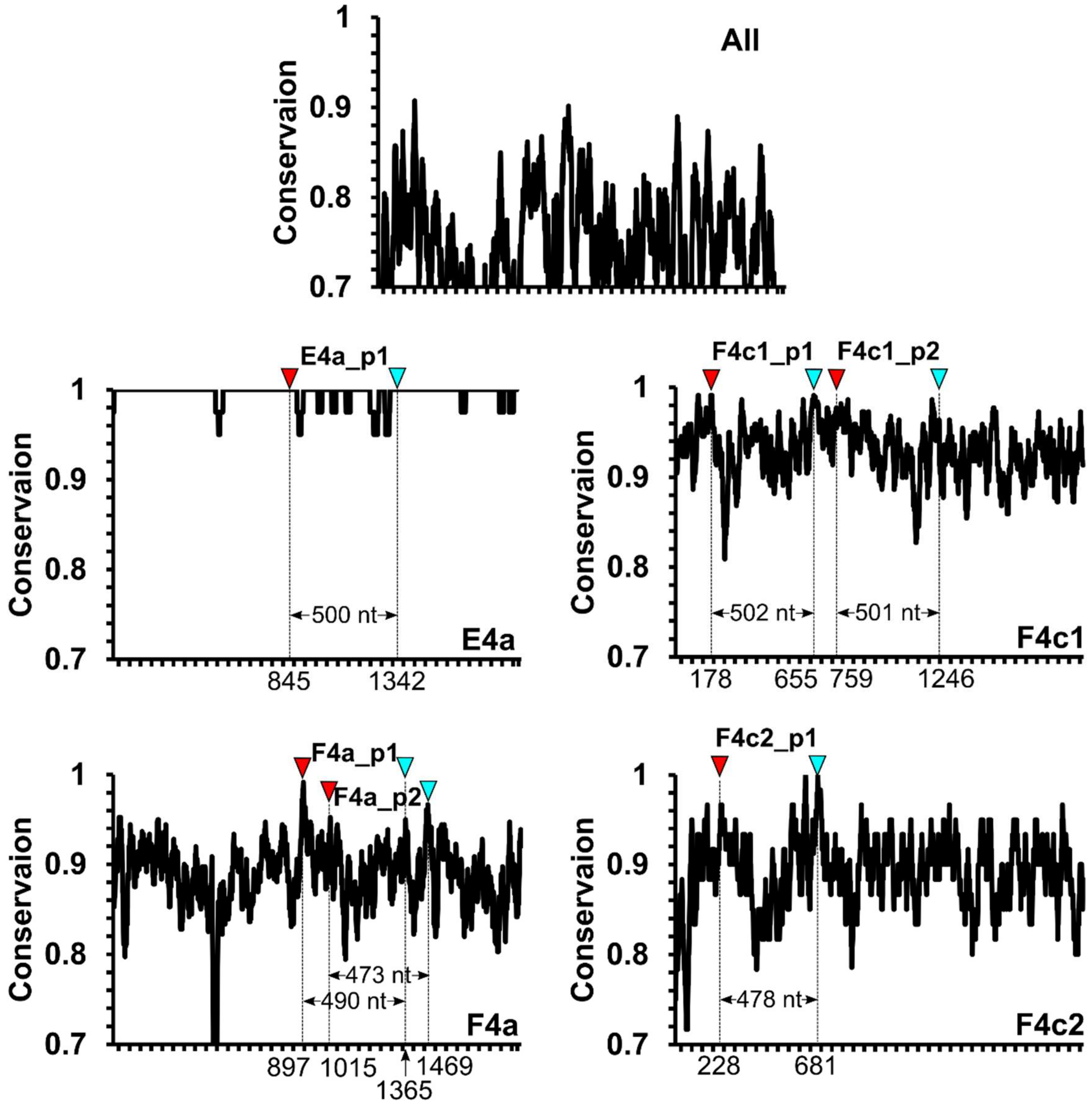

Fig. S3 Conservation of FirmiCODHech nucleotide sequences and primer regions. The average ratio of dominant base to all bases at each position in a 20-base sliding window was calculated and shown in each multiple sequence alignment: "All" indicates that all 34 FirmiCODHech genes were used, and E4a, F4a, F4a1, and F4c2 indicate only the FirmiCODHech genes of each subclade were used. Red and light blue arrow heads indicate the positions of the forward and reverse primers of each new primer set, respectively. The primer set names are shown between the arrow heads and the expected amplicon size is shown on the graph.

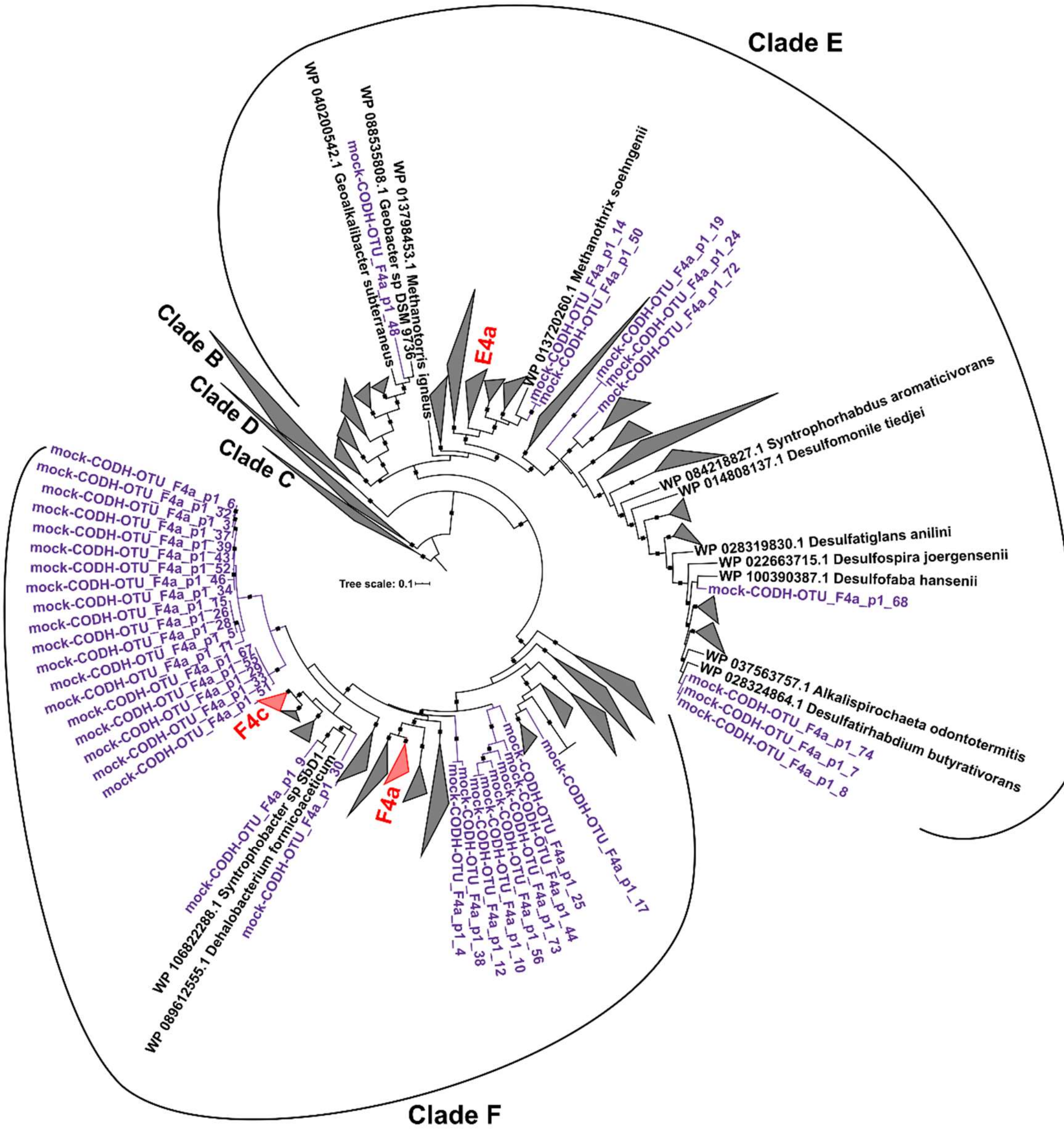

Fig. S4 Phylogeny of 'noisy CODH-OTUs' amplified by the F4a_p1 from the CODHech-mock community sample. The tree was constructed using an alignment of 1,558 CODH proteins and the CODH-OTUs. Major clades, B through F, are indicated. Colors of branches and leaves indicate the following: red, FirmiCODHechs; black, other CODHs; purple, CODH-OTUs. The black squares on the branch indicate >0.8 support by bootstrap values.

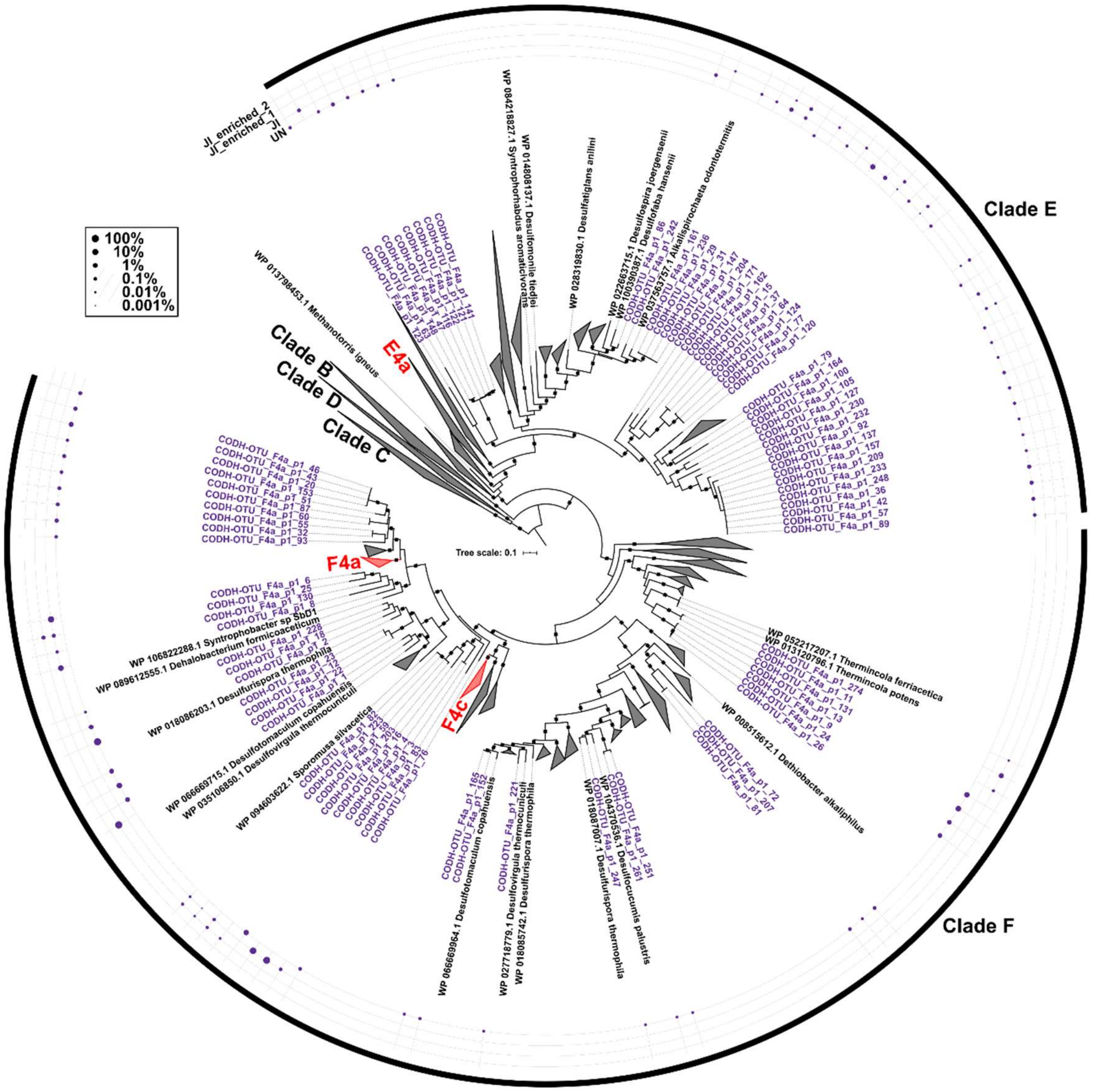

Fig. S5 Phylogeny of 'noisy CODH-OTUs' amplified by the F4a_p1 from the environmental and enrichment samples. The tree was constructed using an alignment of 1,558 CODH proteins and the CODH-OTUs. Major clades, B through F, are indicated. Colors of branches and leaves indicate the following: red, FirmiCODHechs; black, other CODHs; purple, CODH-OTUs. The relative abundance of CODH-OTUs is shown in the bubble plot. The black squares on the branch indicate >0.8 support by bootstrap values.

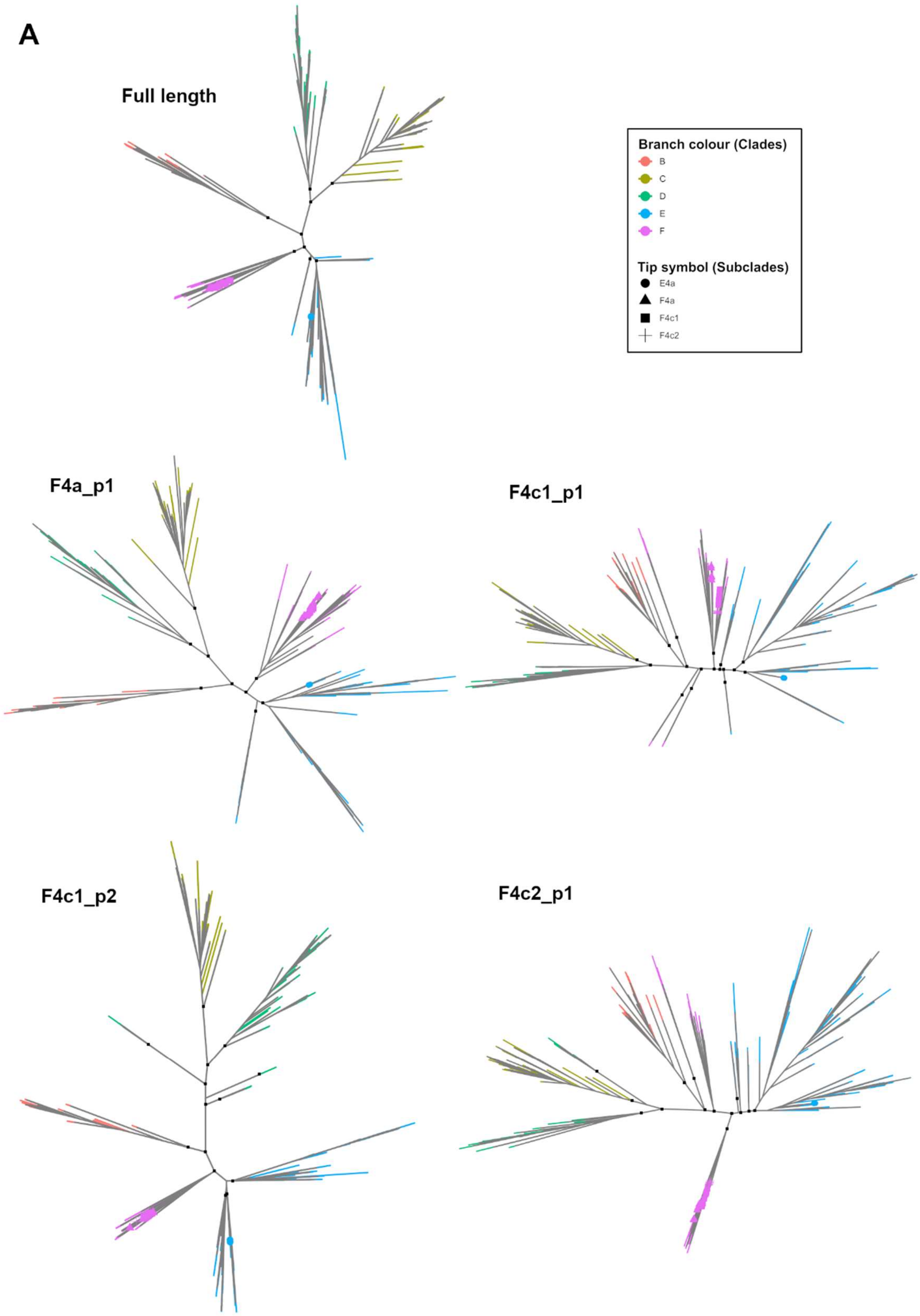
A
Full length
Branch colour (Clades)
B
C
D
E
F
Tip symbol (Subclades)
E4a
F4a
F4c1
F4c2
F4a_p1
F4c1_p1
F4c1_p2
F4c2_p1

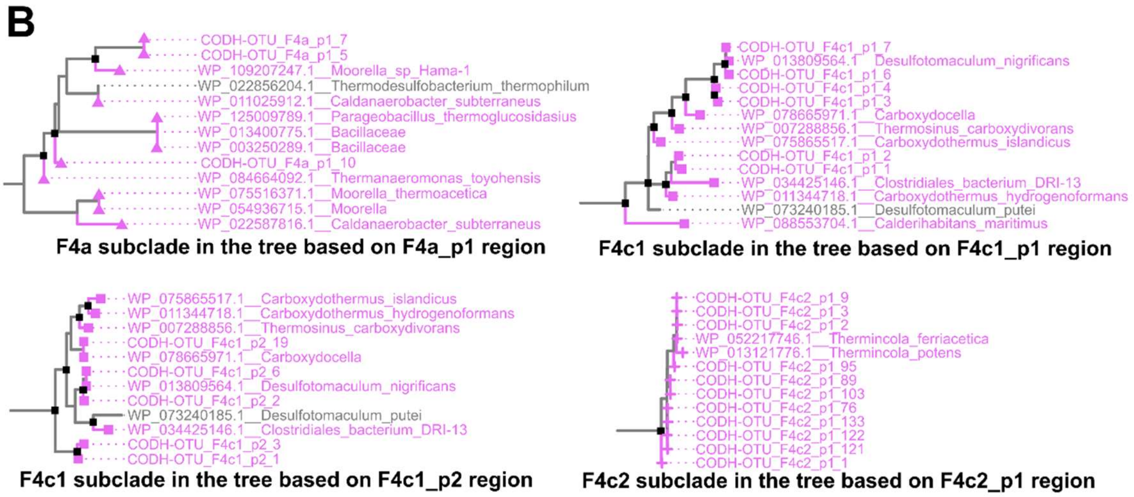

Fig. S6 Phylogeny of the CODH genes and the CODH-OTUs based on different amplification regions of each primer set. Overall unrooted trees (A) and zoom up of FirmiCODHech subclades in each tree (B). The trees were constructed using alignments of 1,558 CODH proteins and the 25 CODH-OTUs obtained from enriched or environmental samples. While the "Full length" tree was constructed from the multiple sequence alignment without trimming (i.e. it is the same tree as Fig. 3), the other trees ("F4a_p1", "F4c1_p1", "F4c1_p2" and "F4c2_p1") were constructed based on the alignments which were trimmed into the CODH-OTU regions of each primer set. Major clades B through F are indicated by different branch colours. FirmiCODHech subclades are indicated by different symbols on the tips. The black squares on the branch indicate >0.8 support by bootstrap values.

Data S1 Nucleotide sequences of the CODH-OTUs that were classified into the FirmiCODHech subclades

>CODH-OTU_F4c1_p1_1
ACCCCAGAGAGGGGTTTGTGGGGCTGATGCTCATACCATTGTTGCGCGAAATTTGATTAGAATGATTGCTGCAGGTGCT
GCTGCCCATTCCGAGCACGGCCGACACATAGCTTTGACTTTATTAGAAGTTGGCGAAGGACATGCACCGGCGTACCGG
ATTAGAGACGAGCAAAAGTTAAGAAATATAGCGGAAAAATTAAACCTGGCACCGGCAGGCAAAAATATAAGACAAGT
GGCAAAAGAAGTGGCTTTAGCTTCCCTGGAAGATTACTCGCGGCAGAAGCAAAACGTACCCTGTACCTGGGCCAAAG
AGACCTTGACCGCTGAGCGGGTGGACAAACTGGCAGAACTGGGGGTTATGCCCCACAATATCGATGCAGTTATCACCG
AGATTATGGGTCGAACTCATGTGGGTTGTGATGCCGATGCAGTCAACGTTTTGCTCGGCGGCT
>CODH-OTU_F4c1_p1_2
ACCCCAGAGAGGGGTTTGTGGGGCTGATGCTCATACCATTGTTGCGCGAAATTTGATTAGAATGATTGCTGCAGGTGCT
GCTGCCCATTCCGAGCACGGCCGACACATAGCTTTGACTTTACTAGAAGTTGGCGAAGGACATGCACCGGCGTACCGG
ATTAAAGACGAGCAAAAGTTAAGAAATATAGCGGAAAAATTAAACTTGGCACCGGCAGGCAAAGATGTAAGACAAGT
GGCAAAAGAAGTGGCTTTAGCTTCCCTGGAAGATTACTCACGGCAGAAGCAAAACGTACCTTGTACCTGGGCCAAAG
AGACCTTGACCGCTGAACGGGTGGACAAACTGGCAGAACTAGGGGTTATGCCCCACAATATCGATGCAGTTATCACCG
AGATTATGGGCCGAACTCATGTGGGTTGTGATGCCGATGCAGTCAACGTTTTGCTCGGCGGCT
>CODH-OTU_F4c1_p1_3
ACCGCAGAGAGGAGTTTGTGGAGCTGATGCCAATACTATTGTGGCTCGAAATTTGGCCAGAATGATTGCTGGAGGTGC
TGCTTCCCATTCCGATCACGGTCGGCACATAGCTTTAACTTTATTAGAAGTTGGCGAAGGTCATGCACCTGCGTACCGG
ATCAAAGATGAGCAAAAGCTAAGAAGTATAGCAGAAAAATTAAACCTGGCACCGGCAGACAAAGACATAAGGCAATT
GGCAAAAGAAGTGGCTCTAGCTTCCCTGGAGGATTACTCGCGTCAGAATAATAGCATACCTTGTAACTGGTCCAAAGCG
ACCATGACTGCTGAACGGGTGGATAAATTGGTAGATCTGGGGGTTATGCCGCATAATATTGATGCTGTTATCGCCGAAAT
TATGGCTCGAACTCATGTAGGTTGTGATGCTGATGCAGCCAATATTTTACTCGGCGGAT
>CODH-OTU_F4c1_p1_4
ACCGCAGAGAGGAGTTTGTGGAGCTGATGCCAATACTATTGTGGCTCGAAATTTGGCCAGAATGATTGCTGGAGGTGC
TGCTTCCCATTCCGATCACGGTCGGCACATAGCTTTAACTTTATTAGAAGTTGGCGAAGGTCATGCACCTGCGTACCGG
ATCAAAGATGAGCAAAAGCTAAGAAGTATAGCAGAAAAATTAAACCTGGCACCGGCAGACAAAGACATAAGGCAAGT
GGCAAAAGAAGTGGCTCTAGCTTCCCTGGAGGATTACTCGCGTCAGAATAATAGCATACCTTGTAACTGGTCCAAAGCG
ACCATGACTGCTGAACGGGTGGATAAATTGGTAGATCTGGGGGTTATGCCGCATAATATTGATGCTGTTATCGCCGAAAT
TATGGCTCGAACTCATGTAGGTTGTGATGCTGATGCAGCCAACATTTTACTCGGCGGAT
>CODH-OTU_F4c1_p1_6
ACCGCAGAGAGGAGTTTGTGGAGCTGATTCCAATACTATTGTGGCTCGAAATTTGGCCAGAATGATTGCTGGAGGTGCT
GCTTCCCATTCCGATCACGGTCGGCACATAGCTTTAACTTTATTAGAAGTTGGCGAAGGTCATGCACCTGCGTACCGGAT
CAAAGATGAGCAAAAGCTAAGAAGTATAGCAGAAAAATTAAACCTGGCACCGGCAGACAAAGACATAAGGCAAGTGG
CAAAAGAAGTGGCTCTAGCTTCCTTGGAAGACTACTCGCGTCAGAATTATAGCATACCTTGTAACTGGACCAAATCGAC
CATGACTGCTGAACGGGTGGATAAATTGGTAGATCTGGGGATTATGCCGCATAATATTGATGCTGTTATCGCCGAAATTAT
GGCTCGAACTCATGTAGGTTGTGATGCTGATGCAGCCAACATTTTACTCGGCGGAT
>CODH-OTU_F4c1_p1_7
ACCGCAGAGAGGAGTTTGTGGAGCTGATGCCAATACTATTGTGGCTCGAAATTTGGCCAGAATGATTGCTGGAGGTGC
TGCTTCCCATTCCGATCACGGTCGGCACATAGCTTTAACTTTATTAGAAGTTGGCGAAGGTCATGCACCTGCGTACCGG
ATCAAAGATGAGCAAAAGCTAAGAAGTATAGCAGAAAAATTAAACCTGGCACCGGCAGACAAAGACATAAGGCAAGT
GGCAAAAGAAGTGGCTCTAGCTTCCTTGGAAGACTACTCGCGTCAGAATTATAGCATACCTTGTAACTGGACCAAATCG
ACCATGACTGCTGAACGGGTGGATAAATTGGTAGATCTGGGGATTATGCCGCATAATATTGATGCTGTTATCGCCGAAAT
TATGGCTCGAACTCATGTAGGTTGTGATGCTGATGCAGCCAACATTTTACTCGGCGGAT
>CODH-OTU_F4c1_p2_1
TGTCAACATCGCGGTTCATGGTCACAACCCTGTGCTTAGTGAGATTATTTGTGATGTAGCGCTGAAGATGAAAGAAGAA
GCTGAGAAAGCCGGGGCTAAAGAAGGTATTAACATAGTGGGTATTTGCTGTACTGGTAATGAGGTAATGATGCGACGGG
GAATTCCTCTGGCTACCAACTACCTGTCCCAGGAACTGGCCATAATTACAGGCGCTGTAGATGCTATGGTCGTAGACGT
GCAGTGTATTATGCCTGCTATTACCAGCTTAGCAGAGTGTTTCCATACAGAAATAATTACCACTATGCCGGTAAACAAAA
TTCCTGGCGCAACGCATGTAGAGTTTCGTGAGGATTCAGCGATAGAAAGTGCGGAGAAAATTGTTGAACTGGCAATTG
AGGCTTTTAAAAGACGGGACAAGCAAAAAGTTAACATTCCTGACTACAAGCAAACAGCCATTGCC
>CODH-OTU_F4c1_p2_2
TGTCAACATTGCGGTTCACGGTCATAACCCGTTACTTAGTGAAATTGTTTGTGATGTAGCGCTGAAGATGAATGAAGAA

GCTAAAAAAGCCGGGGCCAAGGAAGGTATTAACATAGTGGGTATTTGCTGTACGGGTAACGAGGTGATGATGCGACGA
GGGATTCCTCTGGCTACTAACTATCTGTCCCAGGAAATGGCCATAATTACAGGTGCTGTAGATGCTATGGTCGTAGATGT
GCAATGTATTATGCCTGCTATTACCGGCGTAGCTGAGTGTTTCCATACTGAAATAATTACCACTATGCCTGTAAATAAAAT
CACTGGTGCAACTCACATAGAGTTTCGTGAAGACTCGGCCATAGACAGTGCCAGAAAAATTGTTGAGGTGGCAATTGA
TGCCTTTAAAAGACGGGACAGGCGAAAGGTTAACATTCCTGACTACAAGCAAACAGCCATTGCA
>CODH-OTU_F4c1_p2_3
TGTCAACATCGCGGTTCATGGCCACAACCCTGTGCTTAGTGAGATTATTTGTGATGTAGCGCTGAAGATGAAAGAAGAA
GCTGAGAAAGCCGGGGCTAAAGAAGGTATTAACATAGTGGGTATTTGCTGTACTGGTAATGAGGTGATGATGCGACGG
GGAATTCCTCTGGCTACCAACTACCTGTCCCAAGAACTGGCCATAATTACAGGCGCTGTAGATGCTATGGTCGTAGACG
TGCAGTGTATTATGCCTGCTATTACCAGCTTAGCAGAGTGTTTCCATACAGAAATAATTACCACTATGCCGGTAAACAAA
ATTCCTGGCGCAACGCACGTAGAGTTTTGTGAGGATTCAGCGATAGAAAGTGCGGAGAAAATTGTTGAATTGGCAATT
GAGGCCTTTAAAAAACGGGACAAGCAAAAAGTTAACATTCCTGACTACAAGCAAACAGCCATTGCTG
>CODH-OTU_F4c1_p2_6
TGTCAACATTGCGGTTCACGGTCATAACCCGTTACTTAGTGAAATTGTTTGTGATGTAGCGCTGAAGATGAATGAAGAA
GCTAAAAAAGCCGGGGCCAAGGAAGGTATTAACATAGTGGGTATTTGCTGTACGGGTAACGAGGTGATGATGCGACGA
GGGATTCCTCTGGCTACTAACTATCTGTCCCAGGAAATGGCCATAATTACAGGTGCTGTAGATGCTATGGTCGTAGATGT
GCAATGTATTATGCCTGCTATTACCGGCGTAGCTGAGTGTTTCCATACTGAAATAATTACCACTATGCCTGTAAATAAAAT
CACTGGTGCAACTCACATAGAGTTTCGTGAAGACTCGGCCATAGACAGTGCCAGAAAAATTGTAGAACTGGCAATTGA
TGCCTTTAAAAGACGGGACAGGCGAAAGGTTAACATTCCTGACTACAAGCAAACAGCCATTGCA
>CODH-OTU_F4c1_p2_19
TGTTAACATTGCGGTTCACGGTCATAACCCGTTACTTAGTGAAATTATTTGTGATGTCGCGTTGAAAATGAATGAAGAAG
CCAAGAAAGCCGGGGCCAAAGACGGTATTAACATAGTGGGTATTTGTTGTACGGGTAATGAGGTGATGATGCGACGGG
GGATTCCGCTGGCTACTAACTATCTATCCCAGGAAATGGCCATAGTTACAGGCGCTGTAGATGCGATGGTCGTAGATGTG
CAGTGCATTATGCCTGCTCTTACCAGTGTAGCGGAGTGTTTCCATACTGAAGTAATCACCACTATGCCTGTAAATAAAAT
CACTGGCGCCACTCACATAGAGTTTCGGGAAGAATCGGCTGTGGAAAGCGCCAGGAAAATCGTAGAAGTGGCAATTG
AGGCCTTTAAAAGACGGGATAAGCGCAAGGTCAACATTCCTGACTACAAGCAAACAGCCATTGCC
>CODH-OTU_F4c2_p1_1
AGAAACCTGATCAGGGGCATCGCGGCAGGAGCGGCCGCTCATTCCGACCATGGACGGCATATTGCTCTTACTTTGTTAG
AAGTGGCTGAGGGCCATGCCCCGGCGTACTGTATAAAAGATGAGGAAAAATTAATGCACGTCGCCGCCAGGCTGGGCC
TGTCCACCGAAGGCAAAAATGTCAGACAGGTGGCCAAAGAGGTAGCCCTGGCTACGCTGGAAGATTTTTCCCGGCAG
GATTCCAGTATTCCCTGCAGTTGGGCCAGGCAAACGATGACCGCTGAGCGTGCTGAAAAGCTGACTGCCCTTGGCGTC
ATGCCGCATAATATTGATGCTGTTGTAGCAGGCATTATGCACCGAACTCATGTAGGATGTGACGCTGATGCGGTTAACCT
GTTGCTTGGCGGTTTGAAAGGGGCGCTGGCCGACTAT
>CODH-OTU_F4c2_p1_2
AGAAATCTGATCAGGGGCATCGCGGCAGGAGCGGCCGCTCATTCCGACCATGGACGGCATATTGCTCTTACTTTGTTAG
AAGTAGCTGAGGGCCATGCCCCGGCGTACTGCATAAAAGATGAAAAAAAATTAATGAGCGTCGCCGCCAGGCTGGGCC
TGTCCACCGAAGGCAAAAATGTCAGGCAGGTGGCCAAAGAGGTAGCCCTGGCTACGCTGGAGGATTTTTCCCGGCAG
AATTCCAGTATTCCTTGCAGTTGGGCCAGGCAAACCATGACCGCTGAGCGTGCTGAAAAACTGACTGCCCTTGGCGTC
ATGCCGCATAATATTGATGCTGTTGTAGCAGGCATTATGAATCGCACTCACGTAGGATGTGATGCTGATCCGGTTAACCT
GTTGCTTGGCGGTTTGAAAGGGGCGCTGGCCGACTAT
>CODH-OTU_F4c2_p1_3
AGAAACCTGATCAGGGGCATCGCGGCGGGAGCGGCCGCTCATTCCGACCATGGACGGCATATTGCTCTTACTTTGTTAG
AAGTGGCTGAGGGCCATGCCCCGGCGTACTGCATAAAAGATGAAAAAAAATTAATGAGCGTCGCCGCCAGGCTGGGC
CTGTCCACTGAAGGCAAAAATGTCAGGCAGGTGGCCAAAGAGGTAGCCCTGGCTACGCTGGAGGATTTTTCCCGGCA
GAATTCCAGTATTCCTTGCAGTTGGGCCAGGCAAACCATGACCGCTGAGCGTGCTGAAAAACTGACTGCCCTTGGCGT
CATGCCGCATAATATTGATGCTGTTGTAGCAGGCATTATGAATCGCACTCACGTAGGATGTGATGCTGATCCGGTTAACC
TGTTGCTTGGCGGTTTGAAAGGGGCGCTGGCCGACTAT
>CODH-OTU_F4c2_p1_9
AGAAACCTGATCAGGGGCATCGCGGCGGGAGCGGCCGCTCATTCCGACCATGGACGGCATATTGCTCTTACTTTGTTAG
AAGTGGCTGAGGGCCATGCCCCGGCGTACTGCATAAAAGATGAAAAAAAATTAATGAGCGTCGCCGCCAGGCTGGGC
CTGTCCACCGAAGGCAAAAATGTCAGGCAGGTGGCCAAAGAGGTAGCCCTGGCTACGCTGGAGGATTTTTCCCGGCA
GAATTCCAGTATTCCTTGCAGTTGGGCCAGGCAAACCATGACCGCTGAGCGTGCTGAAAAACTGACTGCCCTTGGCGT
CATGCCGCATAATATTGATGCTGTTGTAGCAGGCATTATGAATCGCACTCACGTAGGATGTGATGCTGATCCGGTTAACC
TGTTGCTTGGCGGTTTGAAAGGGGCGCTGGCCGACTAT

>CODH-OTU_F4c2_p1_76
AGAAATCTGATCAGGGGCATCGCGGCAGGAGCGGCCGCTCATTCCGACCATGGACGGCATATTGCTCTTACTTTGTTAG
AAGTGGCTGAGGGCCATGCCCCGGCGTACTGTATAAAAGATGAGGAAAAATTAATGCACGTCGCCGCCAGGCTGGGCC
TGTCCACCGAAGGCAAAAATGTCAGACAGGTGGCCAAAGAGGTAGCCCTGGCTACGCTGGAAGATTTTTCCCGGCAG
GATTCCAGTATTCCCTGCAGTTGGGCCAGGCAAACGATGACCGCTGAGCGTGCTGAAAAGCTGACTGCCCTTGGCGTC
ATGCCGCATAATATTGATGCTGTTGTAGCAGGCATTATGAATCGCACTCACGTAGGATGTGATGCTGATCCGGTTAACCT
GTTGCTTGGCGGTTTGAAAGGGGCGCTGGCCGACTAT
>CODH-OTU_F4c2_p1_89
AGAAATCTGATCAGGGGCATCGCGGCAGGAGCGGCCGCTCATTCCGACCATGGACGGCATATTGCTCTTACTTTGTTAG
AAGTGGCTGAGGGCCATGCCCCGGCGTACTGTATAAAAGATGAGGAAAAATTAATGCACGTCGCCGCCAGGCTGGGCC
TGTCCACCGAAGGCAAAAATGTCAGGCAGGTGGCCAAAGAGGTAGCCCTGGCTACGCTGGAGGATTTTTCCCGGCAG
AATTCCAGTATTCCTTGCAGTTGGGCCAGGCAAACCATGACCGCTGAGCGTGCTGAAAAACTGACTGCCCTTGGCGTC
ATGCCGCATAATATTGATGCTGTTGTAGCAGGCATTATGAATCGCACTCACGTAGGATGTGATGCTGATCCGGTTAACCT
GTTGCTTGGCGGTTTGAAAGGGGCGCTGGCCGACTAT
>CODH-OTU_F4c2_p1_95
AGAAATCTGATCAGGGGCATCGCGGCAGGAGCGGCCGCTCATTCCGACCATGGACGGCATATTGCTCTTACTTTGTTAG
AAGTAGCTGAGGGCCATGCCCCGGCGTACTGCATAAAAGATGAAAAAAAATTAATGAGCGTCGCCGCCAGGCTGGGCC
TGTCCACCGAAGGCAAAAATGTCAGACAGGTGGCCAAAGAGGTAGCCCTGGCTACGCTGGAAGATTTTTCCCGGCAG
GATTCCAGTATTCCCTGCAGTTGGGCCAGGCAAACGATGACCGCTGAGCGTGCTGAAAAGCTGACTGCCCTTGGCGTC
ATGCCGCATAATATTGATGCTGTTGTAGCAGGCATTATGAATCGCACTCACGTAGGATGTGATGCTGATCCGGTTAACCT
GTTGCTTGGCGGTTTGAAAGGGGCGCTGGCCGACTAT
>CODH-OTU_F4c2_p1_103
AGAAATCTGATCAGGGGCATCGCGGCAGGAGCGGCCGCTCATTCCGACCATGGACGGCATATTGCTCTTACTTTGTTAG
AAGTGGCTGAGGGCCATGCCCCGGCGTACTGTATAAAAGATGAGGAAAAATTAATGCACGTCGCCGCCAGGCTGGGCC
TGTCCACCGAAGGCAAAAATGTCAGACAGGTGGCCAAAGAGGTAGCCCTGGCTACGCTGGAGGATTTTTCCCGGCAG
AATTCCAGTATTCCTTGCAGTTGGGCCAGGCAAACCATGACCGCTGAGCGTGCTGAAAAACTGACTGCCCTTGGCGTC
ATGCCGCATAATATTGATGCTGTTGTAGCAGGCATTATGAATCGCACTCACGTAGGATGTGATGCTGATCCGGTTAACCT
GTTGCTTGGCGGTTTGAAAGGGGCGCTGGCCGACTAT
>CODH-OTU_F4c2_p1_121
AGAAATCTGATCAGGGGCATCGCGGCAGGAGCGGCCGCTCATTCCGACCATGGACGGCATATTGCTCTTACTTTGTTAG
AAGTAGCTGAGGGCCATGCCCCGGCGTACTGCATAAAAGATGAGGAAAAATTAATGCACGTCGCCGCCAGGCTGGGCC
TGTCCACCGAAGGCAAAAATGTCAGACAGGTGGCCAAAGAGGTAGCCCTGGCTACGCTGGAAGATTTTTCCCGGCAG
GATTCCAGTATTCCCTGCAGTTGGGCCAGGCAAACGATGACCGCTGAGCGTGCTGAAAAGCTGACTGCCCTTGGCGTC
ATGCCGCATAATATTGATGCTGTTGTAGCAGGCATTATGAATCGCACTCACGTAGGATGTGATGCTGATCCGGTTAACCT
GTTGCTTGGCGGTTTGAAAGGGGCGCTGGCCGACTAT
>CODH-OTU_F4c2_p1_122
AGAAATCTGATCAGGGGCATCGCGGCAGGAGCGGCCGCTCATTCCGACCATGGACGGCATATTGCTCTTACTTTGTTAG
AAGTGGCTGAGGGCCATGCCCCGGCGTACTGTATAAAAGATGAGGAAAAATTAATGCACGTCGCCGCCAGGCTGGGCC
TGTCCACCGAAGGCAAAAATGTCAGACAGGTGGCCAAAGAGGTAGCCCTGGCTACGCTGGAAGATTTTTCCCGGCAG
GATTCCAGTATTCCCTGCAGTTGGGCCAGGCAAACCATGACCGCTGAGCGTGCTGAAAAACTGACTGCCCTTGGCGTC
ATGCCGCATAATATTGATGCTGTTGTAGCAGGCATTATGAATCGCACTCACGTAGGATGTGATGCTGATCCGGTTAACCT
GTTGCTTGGCGGTTTGAAAGGGGCGCTGGCCGACTAT
>CODH-OTU_F4c2_p1_133
AGAAATCTGATCAGGGGCATCGCGGCAGGAGCGGCCGCTCATTCCGACCATGGACGGCATATTGCTCTTACTTTGTTAG
AAGTAGCTGAGGGCCATGCCCCGGCGTACTGTATAAAAGATGAGGAAAAATTAATGCACGTCGCCGCCAGGCTGGGCC
TGTCCACCGAAGGCAAAAATGTCAGACAGGTGGCCAAAGAGGTAGCCCTGGCTACGCTGGAAGATTTTTCCCGGCAG
GATTCCAGTATTCCCTGCAGTTGGGCCAGGCAAACGATGACCGCTGAGCGTGCTGAAAAGCTGACTGCCCTTGGCGTC
ATGCCGCATAATATTGATGCTGTTGTAGCAGGCATTATGAATCGCACTCACGTAGGATGTGATGCTGATCCGGTTAACCT
GTTGCTTGGCGGTTTGAAAGGGGCGCTGGCCGACTAT
>CODH-OTU_F4a_p1_5
CGGCAATGAGGTGATGATGCGGCACGGCATCCCGGCCTGCACCCATTCGGTGAGCCAAGAGATGGCTCTGATTACCGG
GGCGGTAGATGCCATGGTGGTGGATTACCAGTGCGTCATGCCTTCCCTGGCTACGGTGGCAGAGTGCATGGGTACCCGG
CTGATCACCACCATGGAGATCACCAAGATCCCGGGGGCTACCCACGTCAAATTTGCGGAGGAAACGGCCGCAGAGAA
CGCCCGGGAGATCATCCGCCTGGCCATCGAGGCCTTCTCCCGCCGTCGGGGTCGGCCGGTAGACATCCCGCAGTACAG

AGCTAAAGTAGTGGCGGGCTTCTCGGTAGAGGCCATTGTGGCAGCCCTGGCCAAACTGAACGCTCAAGATCCTCTTCA
GCCGTTGCTCGACCAAATCGTCTCCGGCAATATTAGGGGCGTCTGCCTCTTTGCCGGG
>CODH-OTU_F4a_p1_7
CGGCAATGAGGTGATGATGCGGCACGGCATCCCGGCCTGCACCCATTCGGTGAGCCAAGAGATGGCTCTGATTACCGG
GGCGGTAGATGCCATGGTGGTGGATTACCAGTGCGTCATGCCTTCCCTGGCTACGGTGGCAGAGTGCATGGGTACCCGG
CTGATCACCACCATGGAGATCACCAAGATCCCGGGGGCTACCCACGTCAAATTTGCGGAGGAAACGGCCGCAGAGAA
CGCCCGGGAGATCATCCGCCTGGCCATCGAGGCCTTCTCCCGCCGTCGGGGTCGGCCGGTAGACATCCCGCAGTACAG
AGCTAAAGTAGTGGCGGGCTTCTCGGTAGAGGCCATTGTGGCAGCCCTGGCCAAACTGAACGCCCAAGATCCTCTTCA
GCCGTTGCTCGACCAAATCGTCTCCGGCAATATTAGGGGCGTCTGCCTCTTTGCCGGG
>CODH-OTU_F4a_p1_10
GGGCAACGAGGTGATGATGCGGCATGGCATTCCGGCCTGCACCCATTCGGTGAGCCAGGAGATGGCGCTGGTAACCGG
TGCGCTGGATGCCGTGGTAGTGGATTACCAGTGCATCATGCCCTCCCTGGCTACGGTGGCCGAATGCATGGGCACTAAG
CTCATCACTACCATGGAGATCACCAAGATCCCCGGGGCCATCCACATCGACTTTTCAGAGGAGACGGCCGGGGAGAAC
GCCAGACAGATCATCCGCCTGGCCATCGAATCCTTCTCCCGTCGCCGGGGTAAGCCGGTGGACATACCGCCATATAAGA
CCAAGGTGGTGGCGGGCTTCTCCGTGGAAGCCATTCTGAATGCCCTGGCCAAGCTAAACGCCGAGGATCCTTTGAAAC
CCCTGATCGACCAGATCGCCTCCGGCAATATACGAGGAGTCTGCCTCTTCGCCGGG

Data S2 Nucleotide sequences of the genetic regions between the abundant CODH-OTUs and their associated ECH genes which were amplified and determined from each CO-enriched sample. Predicted ORF regions encoding CODH and ECH genes are shown in red and blue font, respectively. Orange and light blue regions indicate CODH-related and ECH-related genes, respectively. Green region indicates ORF overlap. Underlines indicate the regions in which CODH-OTUs were found.

>Je1_F4c1
GAAAGAGCCGAATACCTAAGGGTTATCGCTGATGAAGTAAAAAGAATAGCGTCCCATTTGTTTAATATCGCTATGTTGGC
TCATATCATTGGTTTTGATTCGTTATTTATGCATGTCATGGAGGTACGGGAGATAGTCCAGGATACCAAAGAGACTATTTA
TGGTAATCGGATGGATTTGGCTGCTAATTGTATTGGCGGCGTAAAGTATGACCTTTCCGATGAAAAGATTAAGTACCTGA
TGAAGCAATTAGATAAGCTGAAAAAGCCGTTGGAAGAAATTATTGACATTTATATGAACAACAAATTTGTGCGGGCTCG
CACCGAAGGTGTCGGGATTTTACCTAAAGAAGAAGCCCTCCGCTATGGTGTGGTTGGTCCTGTAGCCAGAGGTTCCGG
TATAGATTATGATGTGCGGGTCAAGAGTCCTTATGCCGCTTATGACAAGATAAAGTTTAATGTTCCTGTAGAAAAGGGTT
GTGATGTTAGAGCAAGAGCCATAGTTAGGCTCAGGGAAATTCAGGAAGCTATCAATATTATTCAGCAATGTCTTAAAGA
ATTGCCGGACGGCCCAACTTGTATAGATTATTTGCCGGAAATACCTGCGGGGGAAGCGGTAGCCAAGTCAGAGGCGCC
GCGGGGAGAACTGATTTATTATCTACGCACAAACGGTTCTGACAAGCCGGAACGGATCAAGTGGCGGGTACCAACTTA
TATGAACTGGGAAGCGCTTCATGTTATGATACCGGGAAATAAAGTAGCTGATATTCCTCTCATAATTAACAGCATTGATCC
CTGTATTTCCTGCACCGAAAAATAGGGAGATGATGCCATGCATGAGTTGGCGATAGTGGATAGTCTTTTCCAGATTATCA
ATACCAAAGTGAAAGAGCACCAAGTTAAAAAAGTCATAAAAGTGAAACTTAAGGTTGGCGAGATGACAGCGGTGGAA
CCTATGACTTTAACTGCGTGTTTTAAGGTTTATGCGCAAGGTACTGTGGCCGAGGGTGCAGAGCTGGTTATTGAGCGGG
TTCCTTTGCAGGGAAAATGTCGGGAATGTACCAATGTGTTTCGGGTTCACAATTACAATTTTCTCTGCCCGCAATGCAA
GAGCAGAGCCATAGATATCGTTGCCGGAGAGGAGCTGTACATTGAGGACCTAGAAGTAGAAAAATGACGATCCCTAGA
AAGGAGGTGAACCGCAATGGCAAGAGCTAAAGAGAATTTCTTTATTTACGCCGATCCCCAAAAATGTATGGGTTGCAA
AAATTGTGAGATAGCCTGTGCTTTGGCTCATGCAGGCTGTGATTTACCAACTGCTGTAACCCAGGGTTTGCAATTACAG
CCGAGAAATTCGGTGGTCCAAGTTGATGGCATGGTTATGCCGATTCAATGCCGACAATGTGAAGATGCGCCTTGTGCCC
ATGCCTGTCCTACAGGAGCAATATTCCAGGAAGGTAGGTTTGTTAAGATTAATGAGAGTAATTGTATTGGCTGTAAAGTT
TGTGCTATGGTTTGTCCTTTTGGAGCTATCATTATAGCAAAAGACGTGAGAGATAAAGATGGTTACAGAACTAAAAATG
GTAAAGCAAGGAAATGTGATCTTTGTTTCAGCAGAAGACAGGATGGCCAGGAAGTATCTTGTGCCTGCGTTGAGGCAT
GTCCTACTAAAGCTATAATGCTGGTAGATTACGAGAGTTATCGTAAGGGACTTATGGAAGAGAGAGGAAAAGAACTTGT
AAGGGCGCATGTAAAAACTAAGGCCTAAGTTGAAATAAGGGGAGTGATTTTATGAATGAAAGGAAAAATTCTTTAGAT
CCTGCTGTTAATTATTTGCTGCCTTTAGCCAAAAAGGCGGGAATTGAAACTGTTTGGGATCGTTTTGCAGCAATGCAGC
CCCAATGTGGTTTCGGAGAGCTGGGAATCTGTTGTCGCATTTGTTGGAAGGGACCTTGTCGTATCGATCCATTTGGCAA
TGGACCCCAGAGAGGGGTTTGTGGGGCTGATGCTCATACCATTGTTGCGCGAAATTTGATTAGAATGATTGCTGCAGGT
GCTGCTGCCCATTCCGAGCACGGCCGACACATAGCTTTGACTTTATTAGAAGTTGGCGAAGGACATGCACCGGCGTACC
GGATTAGAGACGAGCAAAAGTTAAGAAATATAGCGGAAAAATTAAACCTGGCACCGGCAGGCAAAAATATAAGACAA
GTGGCAAAAGAAGTGGCTTTAGCTTCCCTGGAAGATTACTCGCGGCAGAAGCAAAACGTACCCTGTACCTGGGCCAA
AGAGACCTTGACCGCTGAGCGGGTGGACAAACTGGCAGAACTGGGGGTTATGCCCCACAATATCGATGCAGTTATCAC
CGAGATTATGGGTCGAACTCATGTGGGTTGTGATGCCGATGCAGTCAACGTTTTGCTCGGCGGCTTAAAGGGAGCACT
GGCAGATTATACCGGGATGTGTTTGTCAACTGAGTTGTTTGATGCCTTATTTGGTACCCCTGAACCGGTAGTTACCACTG
CCAACCTCGGCGTTTTGAAGGAAAATGCTGTCAACATCGCGGTTCATGGCCACAACCCTGTGCTTAGTGAGATTATTTG
TGATGTAGCGCTGAAGATGAAAGAAGAAGCTGAGAAAGCCGGGGCTAAAGAAGGTATTAACATAGTGGGTATTTGCTG
TACTGGTAATGAGGTGATGATGCGACGGGGAATTCCTCTGGCTACCAACTACCTGTCCCAAGAACTGGCCATAATTACA
GGCGCTGTAGATGCTATGGTCGTAGACGTGCAGTGTATTATGCCTGCTATTACCAGCTTAGCAGAGTGTTTCCATACAGA
AATAATTACCACTATGCCGGTAAACAAAATTCCTGGCGCAACGCACGTAGAGTTTTGTGAGGATTCAGCGATAGAAAGT
GCGGAGAAAATTGTTGAATTGGCAATTGAGGCCTTTAAAAAACGGGACAAGCAAAAAGTTAAC

>Je1_F4c2
GGGCCGAATATCTCCGGGTGATCGCTGATGAGATCAAACGCATCGCCTCACATTTGTTTAATACAGGAATGTTAGCCCAT
ATTATCGGTTTCGATTCATTGTTCATGCACGTAATGGAGTTAAGAGAAATAGTCCAGGATACAAAAGAAACAATTTATGG
AAACCGGATGGACCTGGCGGCCAACAGTATTGGCGGGGTAAGGTACAACCTTACTGAAGAAAAAGGGAAATACCTCC
TGAAAAATTTAGAGCAGCTCAAAAAACCGCTGCAGGAGATTTGCAAAATTTACAGTACCAATCCCCTGGTCAGACTCA
GAACAGAAGGGGTAGGCATATTACCCAAAGAAAAAGCCGTCGAGTATGGAGTAGTCGGGCCTGTCGCCAGGGGTTCC
GGAATCAATTATGATGTACGGGTGAAAAACCCTTACGCAGCATATAACCGGTTGAAGTTTACTGTTCCTGTGGAAACCG
CCGGGGATGTCAGGGCAAGGGCTATTGTAAGGTTAAGAGAAATTGCGGAGTCAATAAGCATCATTGAACAGTGTGTCC
GTGATATGCCCGAGGGACCAACCTGCCTGGATTATTTGCCTGATATACCTGCTGGGGAGGCAGTTGCCAAGTCGGAGGC
GCCGCGGGGGGAACTGCTGTATTATGTGCGTACGGCCAACAGTGATCTGCCTGAACGCATCAAATGGCGCGTACCAAC
TTATATGAACTGGGAAGCTTTGAAAATCATGATTCCAGGGAACAAACTGGCCGATGTTCCGGTTATTATCGGCAGCATTG
ACCCTTGTATTTCCTGCACCGAGAGATAGCCAACCTTAATGCATTAGCTCCGAAGGTTTTCACTATACGGGAGGAAGGG
ATGCCCATGCATGAGGCGGCAATAGTAGAGAGTATTTTCAGTGTCCTCAATGCCCAAATAAAGGAGCACCGGATAAAAA
AAGTATTGAAAGTAAAACTCAGGGTTGGGGAAATGACTGCCATAGAGAATACCACTTTGATAGCCTGTTTCGAAGTTTA
TGCTGAAAATACCGCGGTCCAGGGAGCAGACCTGGTCATCGAGCGGGTTCCGCTCAAGGCAAGGTGTCAGGAGTGCA
AGGAGGAGTTTCCTGTGCTCAATTATAGTTTTCGCTGTTCCCGCTGTGGGGGCATAAATGTACAGGTTATTTCAGGCAG
GGAACTGTACATTGAAAGCATTGAAGCAGAAAAGTTTGAAGAGGAGGTGAATCCCAGTGGCCAAAATTAAGGAGAAC
TCGTTTATTTATGCAGACCCGCAGAAATGCCTGGGCTGCAAAAATTGTGAACTGGCCTGTGCGCTGGCTCACGCGGGTT
GTGACCTGCAAACGGCTGTAGCGGGAAAACGGCAGTTACAGCCGCGCAACTCAGTGGTCAAGGCAGGCGACATGTTT
ATGCCGATTCAGTGCCGGCAGTGTGAAGACGCGCCATGCGCCCAGGCCTGTCCAACGGGAGCTATCAGGCAGGAGGAT
GGATTAGTCAAAATCAACGAACAAAACTGTGTTGGTTGCAAAGTGTGCTCTATGGTTTGCCCTTTTGGAGCCATTGTTG
TGACTCCGGTTCCTAATGCTGAACCAAACCGGAAGACTAAAAGGGGCAAGGCCTTAAAATGTGACCTATGCTTCAGCA
GCGTCCGGAAAGATGGCCAACTAACCTGCGCCTGTGTGGAAGCCTGTCCTCACGCAGGCTGTAATGCTGGTTGATCCT
GAGGGTTACCGGACAAAGATTCTTGAAGCCAGAGCCAAAGAATTGGCCAAGGCTCATTCAGTAGCTAAGCAGAAGCG
GGCATAAAATTGGGGGGAGAAGCGTGAATAAGGATTATAAATTTGTTGACCAGGCAATTGAAGAGCTGCTGCCGAAAG
TCAAGGAGGCAGGATTGGACACGGTTTGGGTCAGGTTCAAAAGCATGCAGCCACAATGCGGATTTGGTGAGCTGGGA
ATTTGCTGCCGTATCTGTTGGAAAGGGCCGTGCCGTATTGACCCCTTTGGTAACGGGCCTCAGCGCGGCATTTGCGGGG
CAGATGCTCACACCATTGTGGCGAGAAATCTGATCAGGGGCATCGCGGCAGGAGCGGCCGCTCATTCCGACCATGGAC
GGCATATTGCTCTTACTTTGTTAGAAGTAGCTGAGGGCCATGCCCCGGCGTACTGCATAAAAGATGAAAAAAAATTAAT
GAGCGTCGCCGCCAGGCTGGGCCTGTCCACCGAAGGCAAAAATGTCAGGCAGGTGGCCAAAGAGGTAGCCCTGGCTA
CGCTGGAGGATTTTTCCCGGCAGAATTCCAGTATTCCTTGCAGTTGGGCCAGGCAAACCATGACCGCTGAGCGTGCTG
AAAAACTGACTGCCCTTGGCGTCATGCCGCATAATATTGATGCTGTTGTAGCAGGCATTATGAATCGCACTCACGTAGG
ATGTGATGCTGATCCGGTTAACCTGTTGC

>Je2_F4c1
AAAGAGCCGAATACCTAAGGGTTATCGCTGATGAAATAAAAAGAATAGCGTCACATTTGTTTAATATCGCTATGTTGGCT
CATATCATTGGTTTTGATTCGTTATTCATGCATGTCATGGAGGTACGGGAGATAGTCCAGGATACCAAAGAGACTATTTAT
GGTAATCGGATGGATTTGGCTGCTAATTGTATTGGCGGCGTAAAGTATGACCTTTCCGATGAAAAGATTAAGTACCTGAT
GAAGCAATTGGATAAACTGAAAAAGCCATTGGAAGAAATTATTGACATTTATATGAACAACAAATTTGTGCGGGCTCGC
ACCGAAGGTGTCGGGATTTTACCTAAAGAAGAAGCCCTCCGCTATGGTGTGGTTGGTCCTGTAGCCAGAGGGTCCGGT
ATAGATTATGATGTGCGGGTCAAGAGCCCTTATGCCGCTTATGACAAGATAAAGTTTAATGTTCCTGTAGAAAAAGGTTG
TGATGTTAGAGCGAGGGCCATAGTTAGGCTCAGGGAAATTCAGGAAGCTATCAATATTATTCAGCAATGTCTTAAAGAA
TTGCCGGACGGCCCAACTTGTATAGATTATTTGCCGGAAATACCTGCGGGGGAAGCAGTAGCCAAGTCAGAGGCGCCG
CGGGGAGAACTGATTTATTATCTACGCACAAACGGTTCTGACAAGCCGGAACGGATCAAGTGGCGGGTACCAACTTAT
ATGAATTGGGAAGCGCTTCATGTTATGATACCGGGAAATAAAGTAGCTGATATTCCTCTCATAATTAACAGCATTGATCCC
TGTATTTCCTGCACCGAAAAATAGGGAGATGATGCCATGCATGAGTTGGCGATAGTGGATAGTCTTTTCCAGATTATCAA
TACCAAAGTGAAAGAACACCAAGTTAAAAAAGTTATAAAAGTGAAACTTAAGGTTGGCGAGATGACAGCGGTGGAAC
CTATGACTTTAACTGCGTGTTTTAAGGTTTATACGCAAGGTACTGTGGCCGAGGGTGCAGAGCTGGTTATTGAGCGGGT
TCCTTTGCAGGGAAAATGCCGGGAATGTACCACTGTGTTTCGGGTTCACAATTACAATTTTCTCTGCCCGCAATGTAAG
AGCAGAGCCATAGATATCGTTGCCGGAGAGGAGCTGTACATTGAGGACTTAGAAGTAGAAAAATGAGGATTCCTAGAA
AGGAGGTGAACCGCAATGGCAAGAGCTAAAGAAAATTTCTTTATTTACGCCGATCCTCAAAAATGTATGGGATGCAAA
AATTGTGAGATAGCCTGTGCTTTGGCTCATGCAGGCTGTGATTTACAAACTGCTGTAACCCAGGGTTTGCGATTACAGC
CAAGAAATTCGGTGGTCCAAGTAGATGGCATGGTTATGCCGATTCAATGCAGGCAATGTGAAGATGCGCCTTGTGCCCA
TGCCTGTCCTACAGGAGCAATATTCCAGGAAGGTAGGTTTGTTAAGATTAATGAGAGTAATTGTATTGGCTGTAAAGTTT
GTGCTATGGTTTGTCCTTTTGGAGCTGTCATTATAGCAACAGACATGAAAGATAAAGATGGTTACAGAACTAAAAATGG
TAAAGCAAGGAAATGTGATCTTTGTTTCAGCAGAAGACAGGATGGCCAGGAAGTATCCTGTGCCTGCGTTGAGGCATG
TCCTACTAAAGCTATAATGCTGGTAGATTACGAGAGTTATCGTAAGAAACTTATGGAAGAGAGAGGAAAAGAACTTGTA
AGGGCGCATGTAAAAACTAAGGCCTAAAGTTGAAATAAGGGGAGTGATTTTATGAATGAAAGGAAAAATTCTTTAGAT
CCTGCTGTTAATTATTTGTTGCCTTTAGCCAAAAAGGCGGGAATTGAGACTGTTTGGGATCGTTTTGCAGCAATGCAGC
CCCAATGTGGTTTCGGAGAGCTGGGAATCTGTTGCCGCATTTGTTGGAAGGGACCTTGTCGTATCGATCCTTTTGGCAA
TGGACCCCAGAGAGGGGTTTGTGGGGCTGATGCTCATACCATTGTTGCGCGAAATTTGATTAGAATGATTGCTGCAGGT
GCTGCTGCCCATTCCGAGCACGGCCGACACATAGCTTTGACTTTACTAGAAGTTGGCGAAGGACATGCACCGGCGTAC
CGGATTAAAGACGAGCAAAAGTTAAGAAATATAGCGGAAAAATTAAACTTGGCACCGGCAGGCAAAGATGTAAGACA
AGTGGCAAAAGAAGTGGCTTTAGCTTCCCTGGAAGATTACTCACGGCAGAAGCAAAACGTACCTTGTACCTGGGCCAA
AGAGACCTTGACCGCTGAACGGGTGGACAAACTGGCAGAACTAGGGGTTATGCCCCACAATATCGATGCAGTTATCAC
CGAGATTATGGGCCGAACTCATGTGGGTTGTGATGCCGATGCAGTCAACGTTTTGCTCGGCGGCTTAAAGGGAGCACT
GGCAGATTATACCGGTATGTGTTTGTCAACTGAGCTGTCTGATGCCTTATTTGGTACCCCTGAACCGGTAGTTACCACTG
CCAACCTCGGCGTTTTGAAGGAAGATGCTGTCAACATCGCGGTTCATGGTCACAACCCTGTGCTTAGTGAGATTATTTG
TGATGTAGCGCTGAAGATGAAAGAAGAAGCTGAGAAAGCCGGGGCTAAAGAAGGTATTAACATAGTGGGTATTTGCTG
TACTGGTAATGAGGTAATGATGCGACGGGGAATTCCTCTGGCTACCAACTACCTGTCCCAGGAACTGGCCATAATTACA
GGCGCTGTAGATGCTATGGTCGTAGACGTGCAGTGTATTATGCCTGCTATTACCAGCTTAGCAGAGTGTTTCCATACAGA
AATAATTACCACTATGCCGGTAAACAAAATTCCTGGCGCAACGCATGTAGAGTTTCGTGAGGATTCAGCGATAGAAAGT
GCGGAGAAAATTGTTGAACTGGCAATTGAGGCTTTTAAAAGACGGGACAAGCAAAAAGT
